# Barriers and facilitators to recruitment, engagement, and retention of underrepresented populations in dementia prevention research: a scoping review^✰^

**DOI:** 10.1016/j.tjpad.2026.100557

**Published:** 2026-04-04

**Authors:** A.F. Rirash, S. Franzen, R. Bourdage, E. Kreuk, N.C. Visser, G.M. Babulal, E. van den Berg, J.M. Papma

**Affiliations:** a Department of Neurology and Alzheimer Center Erasmus MC, Erasmus MC University Medical Center, Rotterdam, the Netherlands; b Department of Medical Psychology, Amsterdam UMC, University of Amsterdam, Amsterdam, the Netherlands; c Amsterdam Public Health Research Institute, Quality of Care & Personalized Medicine, Amsterdam, the Netherlands; d Department of Bioethics & Medical Humanities, Julius Center, UMC Utrecht, Utrecht, the Netherlands; e Department of Neurology, Washington University School of Medicine, St. Louis, MO, USA; f Department of Geriatrics and Alzheimer Center, Erasmus MC University Medical Center, Rotterdam, the Netherlands

**Keywords:** Dementia prevention, Underrepresented populations, Recruitment and retention, Intersectionality, Health equity

## Abstract

Underrepresented populations in dementia prevention research, including minoritized racial/ethnic groups, individuals with lower socioeconomic status, and others facing social and structural disadvantages, are disproportionately affected by dementia risk. This scoping review examined barriers and facilitators to recruitment, engagement, and retention of these populations in Alzheimer’s disease and related dementias (ADRD) prevention studies, synthesizing evidence from both empirical studies and review articles. Guided by PRISMA-ScR and the conceptual structure described by Gilmore-Bykovskyi et al., findings were synthesized from 19 reviews and 53 empirical studies. Findings were interpreted with attention to how overlapping factors—such as ethnicity, age, gender, and structural inequities—may influence study participation. Studies originated primarily from the United States (U.S.). Five key themes were identified: 1) mistrust, 2) stigma and limited research literacy, 3) logistical and financial constraints, 4) communication gaps and lack of team diversity, and 5) systemic-level barriers. Facilitators included culturally tailored outreach, long-term community partnerships, and inclusive study design. Retention strategies remain underreported, and little is known about the non-U.S. context. These findings highlight the need for context-specific, multi-level strategies that address the intersecting barriers faced by underrepresented groups to support equitable participation in dementia prevention research, and ultimately, dementia prevention.

## 1 Introduction

Global projections estimate that the number of people living with dementia will triple from 50 million to 152 million in 2050 [1]. This growing prevalence emphasizes the need to explore opportunities for dementia prevention, especially in the absence of disease-modifying treatments. Amongst all risk factors associated with dementia, 14 are currently considered potentially modifiable, encompassing lifestyle and health-related factors such as cardiovascular health, mental well-being, and physical activity. By tackling these modifiable risk factors, 45 % of dementia cases may be preventable, highlighting the significance of dementia prevention trials [2].

Disparities in dementia risk factors exist across demographic groups; however, some populations have an exponentially higher risk [2]. Individuals from racial and ethnically minoritized (REM) populations—defined here as underrepresented or disadvantaged groups relative to the majority population—face a higher prevalence of potentially modifiable risk factors than the country’s majority population [3]. This is evident for First Nations Australians [4], Black individuals in the United States (U.S.) and the United Kingdom (UK) [5,6], and Hispanic individuals in the U.S [5]. Similarly, individuals with a migration background in Western Europe also face increased risk of developing dementia [7,8], reflecting broader global disparities in dementia risk linked to structural and social determinants of health (S/SDOH) [9,10].

Socioeconomic disadvantages, including lower education and income, are associated with a higher risk of developing dementia [11]. Additionally, chronic exposure to stress, racism, and systemic inequities is identified as a contributor to cognitive decline and dementia, particularly in minoritized populations [2]. To fully understand these disparities, an intersectional perspective is crucial, recognizing how sociodemographic variables such as ethnicity, gender, age, and socioeconomic status (SES) intersect to influence dementia risk. For instance, older adults from REM populations may face multiple disadvantages, including structural racism, limited healthcare access, and ageism [12], all of which interact to amplify their vulnerability to dementia [9,13]. These intersecting factors reinforce syndemic risks, in which the interplay of multiple S/SDOH factors exacerbates cognitive decline [14].

While REM populations are disproportionately affected by dementia risk, they remain underrepresented in dementia prevention and clinical research [15,16], with 75 % of participants in dementia prevention trials being affluent, non-Hispanic White individuals [17]. This underrepresentation raises significant concerns about the generalizability, effectiveness, and equity of dementia prevention efforts [[17], [18], [19]]. Not including those most affected by dementia risk undermines the relevance of prevention strategies and may ultimately reinforce existing health disparities [20].

Understanding the persistence of exclusion in dementia research at large requires a structured approach. Gilmore-Bykovskyi et al. [21] examined the mechanisms that perpetuate the exclusion of REM populations across three intersecting levels of influence: Individual/Interpersonal, Teams and Institutions, and Systems and Structural Norms. At the Individual/Interpersonal level, exclusion can stem from language barriers, transportation challenges, or limited awareness of research opportunities. At the Teams and Institutions level, a lack of cultural competence among researchers and limited remuneration options often hinder engagement. At the Systems and Structural Norms level, broader factors such as funding priorities and inconsistent regulatory or reporting standards perpetuate disparities [21].

Expanding on this conceptual structure [21], barriers to including REM populations in dementia research have been identified across individual, institutional, and systemic levels. For example, mistrust rooted in historical marginalization [17,22], study designs lacking cultural tailoring [[23], [24], [25], [26]], and inconsistent diversity reporting standards [27] are commonly noted obstacles. Facilitators emphasize proactive and context-sensitive strategies, such as multipronged community outreach [26,28], participatory study design [21,29], and sustained investment in community partnerships [27,30]. These insights illustrate how the conceptual structure described by Gilmore-Bykovskyi et al. [21] captures multilevel influences on participation in dementia research more broadly. However, recruitment and engagement strategies are not universally applicable. The effectiveness of an approach often depends on the cultural, linguistic, and structural context of the specific community involved. Tailored approaches are therefore essential, not only to address the specific needs of REM communities, but also to account for variation between countries and regions. Furthermore, the impact of population-attributable factors differs across countries [31], suggesting that the interventions themselves also need to be tailored to specific risk factors.

Several prominent international initiatives have demonstrated the feasibility and value of culturally tailored dementia prevention approaches. Notable examples include the Finnish Geriatric Intervention Study to Prevent Cognitive Impairment and Disability (FINGER), the World-Wide FINGERS network (including U.S. POINTER), and the PREVENT Dementia Program. Each of these programs implements multidomain lifestyle interventions tailored to its respective population. Similarly, the MIND-China study emphasized reducing salt intake as a culturally relevant dietary modification, while the India FINGER trial incorporated yoga as an acceptable form of physical activity [32]. Collectively, these initiatives illustrate that dementia prevention efforts must be tailored to country-specific contexts, population characteristics, and types of research involved. Although culturally tailored interventions are essential, there is a gap in the literature regarding the systematic assessment of the effectiveness on recruitment of these tailored strategies in diverse populations. Moreover, limited attention has been given to retention-specific strategies for REM populations, as little is known about how engagement is maintained once participation commences [24,33].

To inform more inclusive dementia prevention efforts, this scoping review synthesizes evidence from both empirical studies and reviews, offering a meta-level perspective on the barriers and facilitators to REM participation in Alzheimer’s disease and related dementias (ADRD) prevention research. While prior work has identified many of these challenges, this review offers a broader synthesis by integrating findings from empirical studies and reviews, and highlights potential solutions to overcome them. It maps out five key thematic statements and organizes findings inspired by the conceptual structure described by Gilmore-Bykovskyi’ et al. [21], enabling a structured analysis across Individual/Interpersonal, Teams and Institutions, and Systems and Structural Norms levels. By highlighting country-specific challenges and summarizing recruitment, engagement, and retention strategies across different settings, this review aims to identify knowledge gaps and support more inclusive research practices worldwide.

## 2 Methods

### 2.1 Design

We conducted a scoping review, guided by the PRISMA-ScR (Preferred Reporting Items for Systematic Reviews and Meta-Analyses extension for Scoping Reviews) [34] guidelines, to summarize barriers and facilitators to recruitment, engagement, and retention of ethnically diverse populations in ADRD prevention research. As this review was scoping in nature, it was not registered in PROSPERO. The electronic search was conducted in collaboration with the Erasmus MC medical library.

### 2.2 Search strategy

**Fig. 1:**
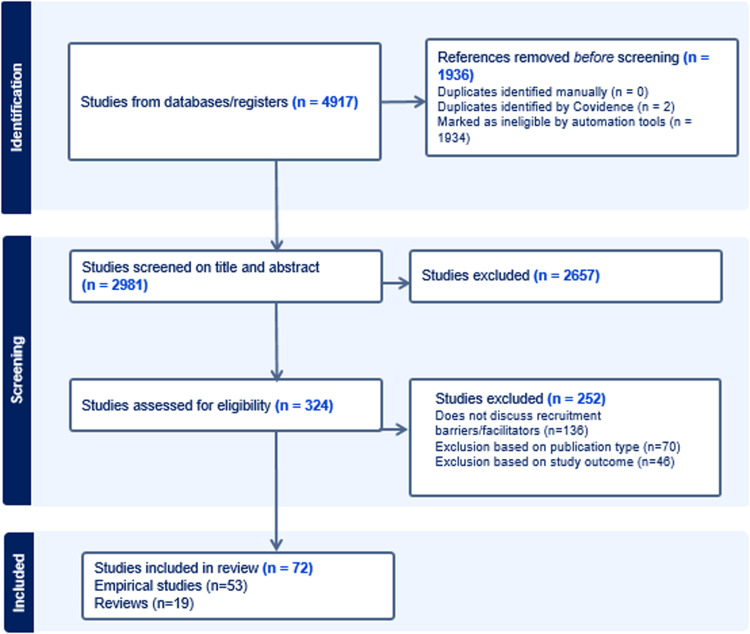
Flow chart of the search strategy. Fig. 1: Flow chart of included papers.

The scoping review was conducted using the Medline, Embase, Web of Science Core Collection, CINAHL, Global Index Medicus (GIM), and Cochrane Library databases. Eligible articles were written in the English language, without restrictions on the year of publication. Unpublished dissertations, book chapters, gray literature, editorials, and conference papers were excluded. The search terms used included various terms related to underrepresented groups, barriers and facilitators, and dementia prevention. For a complete list of search terms and strategy, see Appendix 1. Studies conducted up to July 1, 2025, were considered for inclusion. After removing duplicates, 2981 papers remained for title/abstract screening. After initial screening, 324 papers remained (Fig. 1).

### 2.3 Study selection

#### 2.3.1 Eligibility criteria

Studies were included in the review if they were scoping, narrative, or systematic reviews, or empirical studies investigating best practices for recruitment, engagement, participation, or retention of REM study populations in dementia prevention research. Studies were excluded if they focused only on dementia diagnosis or health-seeking behaviors unrelated to research participation. Moreover, to be eligible for further screening, studies needed to meet the following criteria:

1. The study explicitly investigated or discussed the prevention of Alzheimer’s Disease (AD) or ADRD as a central or a clearly defined secondary focus (e.g., interventions targeting risk reduction or cognitive health maintenance). Studies that only mention ADRD prevention in passing, without targeted analysis, were excluded.
2. The study examined barriers or facilitators of recruitment, engagement, retention, or described strategies to improve these processes in the context of scientific research participation. Studies focused solely on access to healthcare, dementia care, health-seeking behaviors, or cognitive screening/diagnosis and dementia prevalence were excluded, as these settings involve diagnosis-seeking motivations and procedure-specific barriers that are not representative of prevention-focused research. Biomarker or genomic studies in cognitively healthy individuals (*n* = 4) were excluded, given distinct barriers related to invasive procedures (e.g., cerebrospinal fluid (CSF) or blood sample collection) and interpretation of test results.
3. The study was conducted in a cognitively healthy population. Studies focusing on individuals already diagnosed with AD/ADRD, pre-identified *APOE* carriers, or individuals experiencing significant cognitive impairment were not included. Studies focusing on pre-identified *APOE* carriers were excluded, as prior disclosure of genetic risk may influence participants’ motivation, perceived benefits, and engagement with prevention research [35]. These individuals may also have had previous interactions with clinical or scientific research settings to discover their *APOE* status, potentially leading to different participation dynamics than those of cognitively healthy individuals without known biological risk.

#### 2.3.2 Data extraction

Articles were screened at the title/abstract and full-text levels by two independent reviewers (AFR and EK) using Covidence systematic review software. Any conflicts or disagreements that arose during the screening process were resolved through consensus-based discussions. Following the screening process, data extraction was performed independently by AFR and RB. For each included study, key data points were extracted, including the study design, population characteristics, recruitment or retention strategies, reported barriers and facilitators as summarized in the results or discussion sections, primary outcomes, and any relevant conclusions or recommendations. Most included studies reported participants in binary male/female categories, without clarifying whether this referred to sex or gender identity. For consistency, we report these variables as *sex (male/female),* while acknowledging this as a limitation stemming from the reporting in previous studies. Data extraction templates were piloted before full data extraction to ensure consistency in capturing relevant information across studies.

#### 2.3.3 Data analysis

The data were analyzed using a narrative synthesis approach, allowing for the integration of findings from diverse study designs. To support categorization, barriers and facilitators were organized inspired by the three levels of influence described by Gilmore-Bykovskyi et al. [21] This conceptual structure was used to contextualize findings within their primary level of influence, while cross-cutting insights were consolidated into five overarching themes that reflect recurring barriers and facilitators across studies. To avoid double-counting, studies included in both reviews and empirical studies were cross-referenced. The synthesis focused solely on findings summarized in the results and discussion sections, ensuring that only actual study findings were included. Where relevant, findings were interpreted with attention to intersectional factors such as age, gender, and ethnicity, given their combined influence on research participation barriers and facilitators.

## 3 Results

### 3.1 Study characteristics

Out of the 2981 citations identified through the literature search, 2657 were excluded during the title and abstract screening. An additional 252 citations were excluded following a full article review (Fig. 1). Consequently, this review includes 19 reviews and 53 empirical studies. The complete bibliography is provided in Table 1.

**Table 1:** Study characteristics and scoping review results for empirical studies.

Study	Article Title			Field	Study Design	Study Methodology	Target Population	Population Demographics	Barriers to Recruitment	Facilitators to Recruitment	Effectiveness of Recruitment		Facilitators to Retention
Ajrouch et al., 2020	Partnering with Middle Eastern/Arab American and Latino Immigrant Communities to Increase Participation in Alzheimer's Disease Research			ADRD registry	Community research participation report/model	Quantitative research	Middle Eastern-/Arab -Americans and Latinos	Total N: 217 (100 Arab American, 117 Latino) Age: 19+ (52 & 50) Sex: 64 % & 77 % Cognitive Status: Mixed Country: U.S.	No within group diversity or community connections and semantic/language use from translated materials	Communicating with community leaders, rotating recruitment sites, use of translations and interpreters, appropriate setting for events		Effectiveness assessed based on amount of recruits: connecting with community leaders prior to recruitment events led to better recruitment	Community liaison activities to connect with community leaders
Amofa et al., 2023	Health concerns and attitudes towards research participation in a community of rural Black Americans			Dementia screening and clinical research participa-tion	Focus groups + survey	Mixed method research	Black Americans	Total N: 50 Age: 26+ (59) Sex: Not reported Cognitive Status: Not reported Country: U.S.	Time constraints due to roles at home, time burden of research duration, potential side effects, fear of being a lab rat, stigma/shame of others knowing disease, lack of awareness about research opportunities and its benefits, lack of continued communication from research team, fear of getting placebo, access to transportation, lack of appreciation in participating, mistrust and privacy concerns and religious beliefs	Finding a cure, travel reimbursement, altruism, research in group form, continuous involvement of research team in community events and wellbeing, having access to the PI (to improve trust)		Not reported	Not reported
Ashford et al., 2023	Understanding Online Registry Facilitators and Barriers Experienced by Black Brain Health Registry Participants: The Community Engaged Digital Alzheimer’s Research (CEDAR) Study	ADRD registry			Survey	Quantitative research	Black Americans (75 % of respondents)	Total N: 198 Age: 18+ (57) Sex: 83 % Cognitive Status: Not reported Country: U.S.	Time constraints, burdensome effort to join registry, no perceived benefits and technological requirements (having a pc)	Personal interest/gain, altruism, engagement via social media/email and increase diversity in research		Not reported	Learning about personal health, feedback on test results and helping future research to find treatments
Ballard et al., 1993	Recruitment of Black Elderly for Clinical Research Studies of Dementia: The CERAD Experience	ADRD registry			Community research participation report/model	Qualitative report	Black elderly Americans	Total N: 9 consortium sites Age: Not reported Sex: Not reported Cognitive Status: Mixed Country: U.S.	Lack of education and information, economic barriers (transportation fees, health insurance), caregiving cultural differences, difficulties with public transportation, clinic accessibility (location and stigma that it’s for the rich)	Comprehensive and multilingual educational brochures/newsletter, native speakers at gatherings, reimbursement public transport/providing taxi service		Effectiveness assessed based on amount of recruits: familiarity, person-to-person contact, and a relationship of trust led to better recruitment	Not reported
Bardach et al., 2018	Motivators for Alzheimer’s Disease Clinical Trial Participation	AD clinical trial participation			Survey	Quantitative research	Elderly adults	Total N: 87 Age: 54+ (72) Sex: 55 % Cognitive Status: Mixed Country: U.S.	Not reported	Potential to help themselves or loved ones, altruism		Not reported	Engaging various care partners and life companions (including spouses, adult children, partners or friends) throughout the process
Bardach et al., 2020	The Effectiveness of Community-based Outreach Events for the Promotion of African American Research Participation	Recruitment to research center (all types of ADRD research) / AD community events			Community research participation report/model	Quantitative research	African Americans	Total N: 773 Age: Not reported Sex: Not reported Cognitive Status: Not reported Country: U.S.	Time constraints, too young for participation, exclusionary health conditions, lack of interest, need for study partner	Community outreach events are effective		Comparison was made between those who attended more community events vs those who attended less or no event. Participants who attended community events were more likely to participate in research	Not reported
Bardach et al., 2021	Insights From African American Older Adults on Brain Health Research Engagement: “Need to See the Need”	AD research participation			Photovoice (participatory-action research design)	Qualitative research	African Americans	Total N: 21 Age: 35+ (64) Sex: 90 % Cognitive Status: Not reported Country: U.S.	Mistrust, fear of AD, lack of awareness on AD, fear of being mistreated, or receiving the placebo	Dissemination of information, staying in touch, open discussion to address mistrust/concerns/preferences, community organization partnerships, education on research procedures and rights, addressing need for diversity, representative research material images, education of children about the need of brain health		Not reported	Not reported
Bleakley et al., 2022	An elicitation study to understand Black, Hispanic, and male older adults’ willingness to participate in Alzheimer’s-focused research registries	AD recruitment registry			Phone interviews	Qualitative research	Black, White, and Hispanic Americans (20 %,20 %,20 %)	Total N: 60 Age: 49+ (62) Sex: 50 % Cognitive Status: Healthy Country: U.S.	Fear of side effects, privacy concerns, fear of being pressured into a study, fear of confrontation cognitive decline, use of technology, transportation	Personal interest/gain, altruism, remote participation, receiving written information		Not reported	Not reported
Bonner et al., 2017	Trust Building Recruitment Strategies for Researchers Conducting Studies in African American (AA) Churches: Lessons Learned	ADRD recruitment			Interviews	Qualitative research	African Americans	Total N: 4 Age: Not reported Sex: 50 % Cognitive Status: Healthy Country: U.S.	Mistrust, paternalistic attitude of researchers, lack of reciprocity, lack of information, invisibility of PIs	Key persons, presentations, volunteering in community, align research goals with community/church		Not reported	Not reported
Byrd et al., 2011	Recruiting Intergenerat-ional African American Males for Biomedical Research Studies: A Major Research Challenge	Clinical research participation			Survey	Quantitative research	African Americans	Total N: 204 Age: 18+ (not reported) Sex: 100 % male Cognitive Status: Not reported Country: U.S.	Mistrust, time constraints, health reasons	Relative with disease, monetary compensation, altruism, personal gain		Not reported	Not reported
Chao et al., 2011	Recruitment of Chinese American Elders into Dementia Research: The UCSF ADRC Experience	Memory screening			Community research participation report/model	Quantitative research	Chinese Americans	Total N: 453 (80 for survey) Age: (74 non-enrolled; 68 ADRC participants) Sex: Not reported Cognitive Status: Mixed Country: U.S.	Lack of access to research, mistrust, lack of English proficiency, lack of transportation, time constraints	Personal gain, altruism, community outreach events, travel compensation, providing a free lunch		Not reported	Not reported
Custodio et al., 2025	Improving participants' recruitment in dementia-related studies on social media through colloquial language in Lima, Peru			ADRD research	Retrospective case study	Quantitative research	Latinos	Total N: 32 Age: 18+ Sex: Not reported Cognitive status: Healthy Country: Peru	Difficult medical terminology	Social media advertisements, use of colloquial language, culturally tailored recruitment materials		Effectiveness assessed based on amount of recruits: use of colloquial language in advertisements led to better recruitment	Not reported
Darnell et al., 2011	African American Participation in Alzheimer’s Disease Research that Includes Brain Donation	ADRD recruitment + brain donation			Interviews	Qualitative research	African Americans	Total N: 46 Age: 65+ (not reported) Sex: 74 % Cognitive Status: Not reported Country: U.S.	Not reported	Altruism, face to face interviews in a trusted setting, discussion on risks and benefits		Not reported	Establishing and maintaining a relationship of trust
Fritsch et al., 2006	Use of Live Theater to Increase Minority Participation in Alzheimer Disease Research	ADRD recruitment			Community research participation report/model (theater)	Quantitative research	African Americans	Total N: 96 Age: 20+ (not reported) Sex: 83 % Cognitive Status: Not reported Country: U.S.	Not reported	Educational play to improve (AD) research knowledge		Effectiveness assessed based on attendance: reported play as effective	Not reported
Gabel et al., 2023	Remuneration and Recruitment of Study Participants for AD Cohort Studies From the General Public and From Minority Communities	AD cohort studies			Survey	Quantitative research	Hispanic and African Americans (25 %, 25 %)	Total N: 2030 Age: 18+ (not reported) Sex: 52 - 54 % Cognitive Status: Healthy Country: U.S.	Anticipated burden	Monetary compensation		Compared the difference in the amount of compensation ($50 vs. $100) on recruitment effectiveness. No differences were found	Remuneration for participation
Graham et al., 2018	Best strategies to recruit and enroll elderly Blacks into clinical and biomedical research	AD clinical trial participation			Multivariate analysis	Quantitative research	Elderly African Americans, Caucasians, Hispanics and Asians(87 %, 9 %; 1 % and 1 %)	Total N: 3266 Age: 50+ (60) Sex: 69 % Cognitive Status: Mixed Country: U.S.	Not Reported	Health fairs, church and community center collaboration, snowball sampling, recruitment through friend/family		Effectiveness assessed based on percentage of sample recruited by one strategy vs. others: Overall health fairs tailored to their population and advertisements were more effective compared to recruitment via church, family, friend, mass mailing, newspaper, referral and wellness center	Not reported
Grill et al., 2022	Diversifying recruitment registries: Considering neighborhood health metrics	ADRD			Retrospective analysis	Quantitative research	Elderly Adults	Total N: 2837 Age: 18+ (not reported) Sex: 63 % Cognitive Status: Not reported Country: U.S.	Disadvantaged neighborhoods are less well represented in ADRD research	Not reported		Some study sites received a good amount of recruits by direct mail campaigns	Not reported
Hebert et al., 2025	Addressing the knowledge and recruitment gap in Alzheimer's disease and precision medicine research among Native people: an innovative randomized controlled trial			ADRD and Precision Medicine	Randomized controlled trial	Quantitative research	American Indian and Alaska Native	Total N: 812 Age: 40+ (54) Sex: 62 % Cognitive status: Healthy Country: U.S.	Not reported	Culturally tailored recruitment materials, collaboration with community partner, culturally concordant staff		Not reported	Not reported
Hinton et al., 2010	Recruitment of a Community-Based Cohort for Research on Diversity and Risk of Dementia		ADRD recruitment (to AD center)		Community research participation report/model	Quantitative research	Elderly African Americans, Hispanics and Caucasians (44 %; 30 %; 26 %)	Total N: 301 Age: 60+ (77 – Caucasian; 75 - African American; 73 – Hispanic; 76 - other) Sex: 51 % (Caucasian); 71 % (African American); 68 % (Hispanic); 57 % (other) Cognitive Status: Mixed Country: U.S.	Objections from adult children, fear of diagnosis	Bicultural and bilingual staff, at home evaluations, transport provision		Compared referral-based recruitment to recruitment via satellite facilities to conduct active outreach in target community. Outreach recruitment method was more effective.	Not reported
Hughes et al., 2017	African Americans and Clinical Research: Evidence Concerning Barriers and Facilitators to Participation and Recruitment Recommendations		ADRD clinical trial participation		Focus groups	Qualitative research	African Americans / Blacks	Total N: 64 Age: 55+ (66) Sex: 72 % Cognitive Status: Healthy Country: U.S.	Lack of information, fear of research (institutions), mistrust, unknown medication (placebo vs drug), urban legends	Altruism, personal interest/gain, past positive experience with research, familiarity with recruiter, reimbursement, physician referral, personal anecdotes recruiter, education on research, family approach (younger generations to assist in recruitment), meetings to address concerns		Effectiveness based on amount of recruits, most effective were: clergy and churches, radio, social media and television or film	Not reported
Hunsaker et al., 2011	Exploring the Reasons Urban and Rural-Dwelling Older Adults Participate in Memory Research		Memory screening		Focus groups	Qualitative research	Elderly African Americans and Caucasians (65 %; 35 %)	Total N: 55 Age: 65+ (75) Sex:76 % Cognitive Status: Not reported Country: U.S.	Not reported	Personal interest (understanding/insight cognitive functioning), personal gain (access to healthcare services), financial compensation, transport provision, altruism, benefiting family, past positive experience with research, family history with disease		Not reported	Perceived benefits related to research participation may lead to better retention
Jacobsen et al., 2024	Recruitment of a Diverse Community-based Older Adult Cohort for a Longitudinal Aging Study		Aging study		Longitudinal cohort study	Quantitative research	Black participants	Total N: 248 Age: 65+ (71) Sex: 63 % Cognitive status: Healthy Country: U.S.	Using a phone number as means of communication with participants	Continuous relationship building, advertising through printed materials, individual word-of-mouth recruitment (especially for Black males), community events, postcard mailings as a passive strategy combined with active engagement, CAB		Effectiveness assessed based on amount of recruits: postcards, flyers, and word-of-mouth led to better recruitment	Presentations for dissemination
Jefferson et al., 2011	Clinical Research Participation among Aging Adults Enrolled in an Alzheimer’s Disease Center Research Registry		AD clinical trial participation		Survey	Quantitative research	Elderly Caucasians, Black/African Americans and Asians (78 %; 21 %; 1 %)	Total N: 235 Age: 58+ (75) Sex: 60 % Cognitive Status: Mixed Country: U.S.	Time constraints, lack of transportation, travel to city by car required (White respondents), lack of compensation (for time)	Transportation provision, home based visits, financial compensation (more important to non-White respondents)		Not reported	Not reported
Kent et al., 2018	Public Understanding and Opinions of Genetic Research for Alzheimer’s Disease		AD genetic research		Survey including mock AD consent with corresponding knowledge and opinion questions	Quantitative research	Elderly American(White participants and Non-White participants (91 %; 9 %))	Total N: 502 Age: (79) Sex: 58 % Cognitive Status: Mixed Country: U.S.	Mistrust	Personal gain		Not reported	Misperception of personal benefits may negatively affect retention
Lang et al., 2013	African American Participation in Health-Related Research Studies: Indicators for Effective Recruitment		Health related research studies		Survey	Quantitative research	African Americans	Total N: 733 Age 18+ Sex: 60 % Cognitive Status: Mixed Country: U.S.	Mistrust, economic limitations (and lack of health insurance), time constraints	Family history with disease, altruism, financial compensation		Not reported	Not reported
Lee et al., 2023	Using community-based geographical information system (GIS) to recruit older Asian Americans in an Alzheimer's disease study		ADRD clinical research		Longitudinal cohort study	Quantitative research	Korean Americans	Total N: 60 Age: 65+ (not reported) Sex: Not reported Cognitive status: Mixed Country: U.S.	Not reported	Target population analysis to help out in designing tailored recruitment strategies		Not reported	Not reported
Li et al., 2016	Recruiting US Chinese Elders Into Clinical Research for Dementia		ADRD clinical trial participation		Community research participation report/model	Quantitative research	Chinese elderly adults	Total N: 98 Age: 65+ (74) Sex: Not reported Cognitive Status: Mixed Country: U.S.	Not reported	Community lectures, bilingual recruiters, personal gain (insight into cognitive health)		Effectiveness based on amount of recruits per method, most effective was: cooperation with community lecturers compared to advertisements in newspapers	Not reported
Lincoln et al., 2021	Fundamental causes of barriers to participation in Alzheimer’s clinical research among African Americans		AD clinical trial participation		Focus groups	Qualitative research	African Americans	Total N: 44 Age: 50+ (68) Sex: 70 % Cognitive Status: Healthy Country: U.S.	Perception of unequal treatment, mistrust, cultural norms/stigma (within subgroups)	Culturally/ethnically matched research staff, community contact prior to recruitment, dissemination results at original study site		Not reported	Researchers bridging into the community prior to recruiting
Lingler et al., 2022	Mechanisms by Which Cultural-Centric Narrative Influences Interest in ADRD Research Among African American Adults		AD research participation		Community research participation report/model (survey)	Quantitative research	African Americans and Black participants	Total N: 500 Age: 18+ (not reported) Sex: 77 % Cognitive Status: Not reported Country: U.S.	Time burden	Past positive experience with research, personal interest/gain, altruism,		Not reported	Not reported
Mace et al., 2025	Socio-ecological barriers to behavior change-oriented dementia prevention: a qualitative study of healthcare professionals' perspectives		ADRD prevention		Focus group study	Qualitative research	Health care professionals	Total N: 26 Age: Not reported Sex: 73 % Cognitive status: Healthy Country: U.S.	Language barriers, access to information, access to technology, awareness of aging, health care utilization and access, transport, fear of AD, misinformation, fear of negative reaction from patients (perspective of researcher), stringent eligibility criteria, limited time and resources.	Not reported		Not reported	Not reported
Marchant et al., 2025	A multiperspective investigation of the underrepresentation of minoritized ethnic participants in dementia research and proposed strategies to improve inclusive recruitment practices		ADRD research		Focus group study + survey	Qualitative research	REM	Total N: 13 for focus group; 54 for survey Age: Not reported Sex: 85 % for focus group; 69 % for survey Cognitive status: Healthy Country: UK	Limited research literacy, lack of knowledge of disease, stigma dementia, lack of awareness research opportunities, limited digital literacy, language barrier, time constraints, institutional racism, stringent eligibility criteria, lack of cultural congruence of researchers, location of research time, lack of time and funding of research institutions	Offline recruiting materials like paper, recruitment at trusted sites, social media advertisements, key persons, culturally sensitive recruitment materials, co-research with community, receiving dissemination through trusted platforms, quota diverse participants, providing cultural competence training		Not reported	Not reported
Marquez et al., 2022	Increasing engagement of Hispanics/Latinos in clinical trials on Alzheimer’s disease and related dementias	ADRD clinical trial participation			Focus groups	Qualitative research	Hispanics/ Latino/a’s	Total N: 193 Age: 18+ (49) Sex: 69 % Cognitive Status: Healthy Country: U.S.	Limited knowledge (about ADRD or research), stigma AD, fear of diagnosis	Multilingual information, education on research and risk/benefits, collaborations with trusted organizations, altruism, bicultural/bilingual researchers, visibility researcher in community, transportation provision, at home visits, flexible times, appropriate incentives (money/knowledge/other medical help), testimonials enrolled participants		Not reported	Not reported
Mindt et al., 2023	The Community Engaged Digital Alzheimer’s Research (CEDAR) Study: A Digital Intervention to Increase Research Participation of Black American Participants in the Brain Health Registry	ADRD registry			Community research participation report/model (digital)	Quantitative research	African Americans/Black Americans, mixed Americans, Latinos	Total N: 349 Age: 18+ (58) Sex: 85 % Cognitive Status: Mixed Country: U.S.	Not reported	Social media for dissemination and discussion (culturally informed engagement materials), testimonials enrolled participants, idea of increasing diversity, mailings/blogs on educational sources, monetary compensation, family history disease		Not reported	Not reported
Moukarzel et al., 2025	Tailoring implementation strategies for the healthy actions and lifestyles to Avoid Dementia or Hispanos y el ALTo a la Demencia Program: Lessons learned from a survey study	ADRD prevention			Cross-sectional study + focus group	Mixed methods research	Latino	Total N: 157 Age: 50 – 85 (64) Sex: 70 % Cognitive status: Healthy Country: U.S.	Access to technology, time commitment, health issues	Hearing about the study via doctor or family member or friend, wearables for feedback, key persons		Effectiveness assessed based on amount of recruits: hearing about the study via doctor/friend/family led to better recruitment	Not reported
Neffa-Creech et al., 2023	Understanding Barriers and Facilitators to Signing Up for a Mobile Responsive Registry to Recruit Healthy Volunteers and Members of Underrepresented Communities for Alzheimer’s Disease Prevention Studies	ADRD registry			Focus groups + survey	Mixed method research	Black and Hispanic participants	Total N: 39 (focus group), 1010 (survey) Age: 45+ (focus group); (61 - survey) Sex: 49 % (focus group); 61 % (survey) Cognitive Status: Healthy Country: U.S.	Privacy concerns, mistrust, no personal benefit (lack of knowledge on prevention of AD), not comfortable using mobile device to sign up	Family history with disease, altruism, financial compensation, idea of increasing diversity, concise language		Not reported	Not reported
Nissim et al., 2024	Age-Specific Barriers and Facilitators to Research Participation Amongst African Americans in Observational Studies of Memory and Aging	ADRD research			Cross-sectional study + focus group	Mixed methods research	African Americans	Total N: 240 Age: 18+ (not reported) Sex: 80 % Cognitive status: Healthy Country: U.S.	Invasiveness study, inflexible study times, requirement to complete tests, transport, time constraints, lack of digital literacy, systemic racism, duration of study, mistrust	Remote testing, access to test results, flexible study times, financial compensation, providing transport		Not reported	Not reported
Pugh et al., 2022	Beliefs, Understanding, and Barriers Related to Dementia Research Participation Among Older African Americans	ADRD prevention intervention recruitment			Focus groups	Qualitative research	African Americans	Total N: 51 Age: 55+ (68) Sex: 77 % Cognitive Status: Healthy Country: U.S.	Fear of receiving placebo, idea of being a guinea pig, (long) study duration, fear of invasive procedures, mistrust	Non-invasive studies, monetary compensation, transportation provision, transparent informed consent process, education on disease/research, visibility researcher in community		Not reported	Community engagement
Raman et al., 2021	Disparities by Race and Ethnicity Among Adults Recruited for a Preclinical Alzheimer Disease Trial	AD trial			Cross-sectional study	Quantitative research	Black, Hispanic, and Asian participants	Total N: 5945 Age: 65+ (72) Sex: 59 % Cognitive Status: Healthy Country: North America	Ineffectiveness of centralized recruitment efforts, strict eligibility criteria (MMSE scores - Hispanics; CDR criteria - Blacks; Logical Memory scores - Asians, Blacks and Hispanics)	On site recruitment, community outreach recruitment, local earned media		Effectiveness based on amount of recruits per method, most effective was: recruitment via local sites and local media.	Not reported
Ramirez et al., 2025	Overcoming Barriers to Latino Participation in Alzheimer's Disease Research	ADRD clinical research			Longitudinal cohort study	Quantitative research	Latino participants	Total N: 155 Age: 60+ (73) Sex: 56 % Cognitive status: Healthy Country: U.S.	Low awareness about AD, low awareness about research opportunities, long distance to study site, transport, financial burden (gas, parking, etc.), research procedures, mistrust	Multilingual social media advertisements, remote study site in area, offer transportation service, incentive instead of reimbursement, community partner collaboration, multilingual educational content		Effectiveness assessed based on amount of recruits compared to previous years: relaxation of requirements of invasive research procedures as a precondition for participation led to better recruitment	Not reported
Sajatovic et al., 2023	A Randomized Prospective Survey Targeting Knowledge, Barriers, Facilitators and Readiness to Participation in Dementia Research	ADRD research			Randomized prospective survey	Quantitative research	African Americans	Total N: 242 Age: 18+ (58) Sex: 75 % Cognitive status: Healthy Country: U.S.	Not reported	Culturally tailored education, CAB		Not reported	Not reported
Sajatovic et al., 2025	A Randomized Prospective Survey to Understand Readiness for Participation in Dementia Research Studies Across Diverse Communities: Une enquete prospective a repartition aleatoire visant a comprendre la disposition a participer a des etudes de recherche sur la demence dans diverses communautes	ADRD research			Randomized prospective survey	Quantitative research	Latino participants	Total N: 184 Age: 18+ (40) Sex: 57 % Cognitive status: Healthy Country: U.S.	Not reported	Having greater financial security increases chance of research participation, having a college education increases chance of research participation, CAB, culturally tailored education		Not reported	Not reported
Scharff et al., 2010	More than Tuskegee: Understanding Mistrust about Research Participation	AD and cancer related trials			Focus groups	Qualitative research	African Americans	Total N: 70 Age: 18+ (53) Sex: Not reported Cognitive Status: Not reported Country: U.S.	Mistrust, no personal benefit, misinformation, lack of dissemination, limited knowledge about research opportunities, privacy concerns, logistical concerns	Small group information sessions, concise language dissemination		Not reported	Not reported
Schnieders et al., 2013	Incentives and Barriers to Research Participation and Brain Donation Among African Americans	AD research participation (and brain donation)			Educational interview	Qualitative research	African Americans	Total N: 91 Age: 65+ (73 - enrolled; 75 - not enrolled) Sex: 74 % - enrolled; 77 % - not enrolled Cognitive Status: Not reported Country: U.S.	Fear of invasiveness, transportation, privacy concerns, time burden, mistrust, lack of diversity	Personal benefit, altruism, personal interest, monetary compensation, increase diversity, provision transportation		Not reported	Reasons for withdrawing from the study were failing health and excess time commitment
Sewell et al., 2021	Research Attitudes and Interest Among Elderly Latinxs: The Impact of a Collaborative Video and Community Peers	ADRD research participation			Survey	Quantitative research	Predominantly elderly Hispanic participants	Total N: 178 Age: 74 Sex: 81 % (cohort 2, cohort 1 is not reported) Cognitive Status: Not reported Country: U.S.	Not reported	Educative video (increased trust, safety, and volunteerism), use of role models and community advisory board, meeting (Q&A) to address concerns, lay educators		Compared participants who saw video to those who did not: those who saw the video were more willing to participate in research	Not reported
Shaw et al., 2022	Recruitment of Older African Americans in Alzheimer’s Disease Clinical Trials Using a Community Education Approach	AD clinical trial participation			Community research participation report/model	Quantitative report	African Americans	Total N: 66 Age: 21+ (not reported) Sex: 91 % Cognitive Status: Not reported Country: U.S.	Not reported	Community lectures (on research), collaboration with community organizations		Not reported	Not reported
Shaw et al., 2024	Using Focus Groups to Explore Older Black Men's Perception of Dietary Interventions	ADRD prevention			Focus group + survey	Mixed methods research	Black/African Americans	Total N: 10 Age: 55+ (not reported) Sex: 0 % Cognitive status: Healthy Country: U.S.	Lack of knowledge about AD, financial burden	Culturally tailored intervention, culturally tailored communication and education (e.g., via gospel), competitive driven approach, key persons		Not reported	Not reported
Sim et al., 2024	Understanding engagement in diet and dementia prevention research among British South Asians: a short report of findings from a patient and public involvement group	ADRD prevention			Public and patient involvement, roundtable discussion	Qualitative research	British South Asians	Total N: 26 Age: Not reported Sex: 92 % Cognitive status: Healthy Country: UK	Lack of knowledge about AD prevention, stigma of dementia, lack of cultural adaptation intervention, lack of time, language barrier, financial burden, mistrust, privacy concerns	Collaboration with trusted organizations, word-of-mouth recruitment, key persons, local research site, community events, culturally matched research staff, culturally appropriate advertisements		Not reported	Not reported
Struble et al., 2023	Including Socially Isolated Black, Older Old Adults (Aged 80 and Above) with and without Mild Cognitive Impairment in a Clinical Trial: Recruitment Strategies and Perspectives	ADRD research			Longitudinal cohort study	Quantitative research	African Americans	Total N: 186 Age: 75+ (not reported) Sex: Not reported Cognitive status: Mixed Country: U.S.	Regional regulations, variations in institutional policies, diverse community preferences	Involving family in recruitment, culturally tailored recruitment materials		Effectiveness assessed based on amount of recruits comparing two research sites: involving family in recruitment has potential	Time commitment, family commitment, internet issues were reported as barriers to retention
Walker et al., 2024	Recruiting a prospective community cohort to study Alzheimer's disease and structural and social determinants of health among adults racialized as Black: The ARCHES cohort	ADRD research			Longitudinal cohort study	Quantitative research	Black/African Americans	Total N: 238 Age: 45+ Sex: 79 % Cognitive status: Healthy Country: U.S.	Solely use of passive recruitment, process requires time dedication and resources of research team, ‘helicopter research’	Snowball sampling, recruitment via established registries, community health fairs, culturally tailored recruitment materials, CAB, target population analysis, presentation to raise awareness on AD/research, community outreach events (like brunch), combining recruitment methods		Effectiveness assessed based on amount of recruits: snowball sampling provided highest yield for participants	Not reported
Waterink et al., 2025	Evaluation of efficiency and effectiveness of different recruitment strategies for the FINGER-NL multidomain lifestyle intervention trial via the Dutch Brain Research Registry	Brain health registry			Longitudinal cohort study	Quantitative research	Elderly general population	Total N: 1008 Age: 18+ (66) Sex: 68 % Cognitive status: Healthy Country: the Netherlands	Not studied	National television advertisement, Facebook advertisement		Effectiveness assessed based on amount of recruits: national television advertisement and Facebook advertisement yielded the most recruits	Not reported
Williams et al., 2011	An Interdisciplinary Outreach Model of African American Recruitment for Alzheimer’s Disease Research	AD research participation			Community research participation report/model	Quantitative report	African Americans	Total N: Not reported Age: Not reported Sex: Not reported Cognitive Status: Mixed Country: U.S.	Not reported	Social marketing approach, collaboration community organizations (and clergy), presentations at churches, health care provider training (for referrals), community advisory board		Not reported	Sustained interactions with participants and their families, and developing lasting partnerships with community organizations and health professionals
Zhai et al., 2022	Perceptions and Beliefs of Memory Loss and Dementia Among Korean, Samoan, Cambodian, and Chinese Older Adults: A Cross-Cultural Qualitative Study	ADRD research participation			Focus groups	Qualitative research	Cambodian, Chinese, Korean, and Samoan elders	Total N:62 (14 Cambodian, 21 Chinese, 14 Korean, 13 Somoan) Age: 50+ (72) Sex: 68 % Cognitive Status: Not reported Country: U.S.	Language barrier (communication), limited knowledge (about ADRD), concerns about own abilities, logistical concerns (transportation), financial concerns, lack of accessibility	Multilingual/cultural learning materials, intervention in group setting (instead of one-on-one), education ADRD/research, bilingual researchers		Not reported	Not reported
Zhou et al., 2017	African Americans are less likely to enroll in preclinical Alzheimer’s disease clinical trials	AD research participation			Interviews + survey	Mixed method research	African Americans	Total N: 125 (47 African Americans, 78 Whites) Age: 65+ (74 Whites; 72 African American) Sex: 65 % (Whites); 79 % (African American) Cognitive Status: Healthy Country: U.S.	Fear of study invasiveness, requirement of study partner, study location, fear of receiving placebo	(Monetary) compensation		Not reported	Not reported
Note: Age is reported as age range (mean age); Sex is reported as % female participants; cognitive status reported as either healthy or mixed meaning healthy and patient participants; AD = Alzheimer’s Disease; ADRD = Alzheimer’s Disease and Related Dementias; CAB = community advisory board; PI = principal investigator; UK = United Kingdom; U.S. = United States													
**Study**	**Article Title**	**Field**			**Study Methodology**	**Target Population**	**Population Demographics**	**Barriers to Recruitment**		**Facilitators to Recruitment**		**Effectiveness of Recruitment**	**Facilitators to Retention**
Ballard et al., 2010	Challenges and Opportunities: Recruitment and Retention of African Americans for Alzheimer's disease Research: Lessons Learned	AD(RC) research recruit-ment			Review + report	African Americans	Total k: Not reported Age: Not reported Sex: Not reported Cognitive Status: Mixed Country: U.S.	Mistrust, use of language, time constraints, lack of reciprocity (no dissemination), one size fits all approach		Altruism, community organizations collaborations, Q&A meetings, cultural sensitive recruitment materials, social events, CAB in recruitment, transparency research staff		Effectiveness based on amount of recruits: reported as successful	Retention considered successful based on percentage of retention on follow-up, which was 81 %.
Barnes & Bennett, 2014	Alzheimer’s Disease In African Americans: Risk Factors And Challenges For The Future	AD clinical research recruitment			Narrative review	African Americans	Total k: Not reported Age: Not reported Sex: Not reported Cognitive Status: Mixed Country: U.S.	Mistrust, transportation, time constraints (caregiving)		Communication on research goals and benefits, networking with community gatekeepers, constant physical presence in community, maintaining frequent contact, dissemination of findings, culturally matched research staff, culturally sensitive recruitment material, health education		Not reported	Not reported
Dabiri et al., 2024	Examining the Role of Community Engagement in Enhancing the Participation of Racial and Ethnic Minoritized Communities in Alzheimer's Disease Clinical Trials; A Rapid Review	ADRD clinical trials and observational studies			Rapid review	Ethnic minorities	Total k: 49 Age: Not reported Sex: Not reported Cognitive Status: Not reported Country: Mostly U.S.; 1 paper UK	Knowledge gaps about ADRD, mistrust, stigma around dementia, fear of receiving diagnosis, cultural stigma regarding participation (for Latino participants), transportation, inflexible research times, lack of time, language barriers		Partnering with faith based organizations, CAB, key persons, sustained presence in community, organizing health fairs, bilingual research team members, cultural appropriate education materials, diversity of study staff, addressing community needs, providing transportation, flexible schedule		Not reported	Not reported
Dilworth-Anderson et al., 2005	Recruitment Strategies for Studying Dementia in Later Life among Diverse Cultural Groups	AD research recruitment			Narrative review	Not specified (ethnic minorities)	Total k: Not reported Age: Not reported Sex: Not reported Cognitive Status: Mixed Country: U.S.	Mistrust, transportation, no rapport with clinic staff, cultural stigma of dementia, time constraints (caregiving)		Learn about? subcultures within communities, networking with community gatekeepers, frequent up-front meetings, transportation provision, culturally sensitive materials, Q&A (1 on 1) sessions, physical presence in community, community leaders to disseminate findings, attend community events, assure confidentiality, take time (research staff), CAB		Not reported	Not reported
Epps et al., 2024	Synthesizing Best Practices to Promote Health Equity for Older Adults Through Community-Engaged Research	ADRD research			Narrative review	Ethnic minorities, LGBTQIA+ populations	Total k: N/A Age: Not reported Sex: Not reported Cognitive Status: Mixed Country: U.S.	Helicopter (‘fly-by’) recruitment		Community engaged research, building relationships with organizations, appropriate allocation of financial resources and allow time for building relationships		Not reported	Not reported
Gilmore-Bykovskyi et al., 2019	Recruitment and retention of under-represented populations in Alzheimer’s disease research: A systematic review	ADRD research recruitment			Systematic review	Not specified (ethnic minorities and/or disadvant-aged socioeconomic background)	Total k: 22 Age: Not reported Sex: Not reported Cognitive Status: Mixed Country: 21 U.S.; 1 U.K.	Mistrust, fear of complications, insufficient information, fear of invasive procedures, transport, unaware of benefits/accessib-ility, financial barriers, time constraints (caregiving), lack of guidance for replication of recruitment strategies (for researchers)		Altruism, understanding research procedures and goals, monetary compensation, transportation provision, culturally/linguistically matched researchers, dissemination of research, desire to help family, meeting in familiar location		Methods to assess effectiveness varied. The most common method was to track new enrollments by evaluating total number of new participants for the targeted minority group.	Studies often did not specify whether specific activities were related to recruitment or retention
Godbole et al., 2022	Assessing Equitable Inclusion of Underrepresented Older Adults in Alzheimer’s Disease, Related Cognitive Disorders, and Aging-Related Research: A Scoping Review	ADRD research recruitment			Scoping review	Underrepresented groups (ethnic minorities, disadvantaged socioeconomic, rural populations, groups with disabilities, LGBTQ communi-ties)	Total k: 436 Age: 55+ Sex: Not reported Cognitive Status: Mixed Country: U.S.	Mistrust, fear of complications, stigma dementia, insufficient information about study/procedures, communication language and appropriateness, informed consent understanding, transportation, geographic accessibility, time constraints (work hours), financial barriers, seeing no relevance/benefit in study, lack of collection and reporting variables (researchers)		Ethnically matched research staff, transparency/education on research procedures, community based partners, bilingual/bicultural staff, appropriate language use, frequent personal contact (calling + mail), transportation provision, outreach and recruitment in familiar locations, flexible research times, rotating locations, pilot recruitment methods, open randomization instead of blind, monetary compensation, use of personal stories		Most effective is: addressing barriers concerning participant attitudes and perceptions	Maintaining positive relationships between researchers and participants, updating participants on study progression and results, maintaining contact with participants, care partners and community-based partners
Indorewella et al.,2021	Modifiable Barriers for Recruitment and Retention of Older Adults Participants from Underrepresented Minorities in Alzheimer’s Disease Research	AD clinical research recruitment			Narrative review	Elderly and ethnic minorities	Total k: N/A Age: Not reported Sex: Not reported Cognitive Status: Mixed Country: U.S.	Strict eligibility criteria, need for study partner, study partner burden, fear of complications, logistical barrier (time burden, financial barrier, length of study, lack of transport, lack of awareness of research opportunity, lack of representation study staff, mistrust, stigma disease/cultural beliefs		Transparency eligibility criteria and rationale, flexible times meetings, remote participation, in-home visits, monetary compensation, addressing barriers by staff, education on research knowledge, collaboration community based care providers, networking with community key persons, ethnically/linguistically matched researchers		Not reported	Not reported
Lazaar et al., 2025	Diversity in United States Dementia Prevention Trials: An Updated Systematic Review of Eligibility Criteria and Recruitment Strategies	AD prevention research			Systematic review	Ethnic minorities	Total k: 44 Age: 45+ (not reported) Sex: Not reported Cognitive Status: Healthy Country: U.S.	Stringent eligibility criteria, inadequate reporting of ethnicity, passive recruitment, recruitment locations, lack of awareness about dementia prevention		Tracking and reporting inclusion rates per ethnicity, alternative recruitment locations, active recruitment, collaboration with community partners, diversity within research team		Not reported	Not reported
Massett et al., 2021	Facilitators, Challenges, and Messaging Strategies for Hispanic/Latino Populations Participa-ting in Alzheimer’s Disease and Related Dementias Clinical Research: A Literature Review	ADRD clinical research recruitment			Narrative review	Latino/a participants	Total k: 210 Age: Not reported Sex: Not reported Cognitive Status: Mixed Country: U.S.	Lack of knowledge on ADRD/research, limited cultural/linguistic competency research staff, language barrier, low health literacy levels (preventing from accessing healthcare), privacy concerns (immigration status), mistrust, logistical barriers (time constraints (roles in family), financial costs of childcare, gas and transportation), strict eligibility criteria		Positive relationship with healthcare provider, altruism, involving family in decision making, personal interest, diversification study staff, culturally sensitive communication, recruitment videos, word-of-mouth recruitment		Not reported	Holding baseline interviews in a convenient location, conducting brief follow-up interviews through the phone instead of in-person, and providing participants the opportunity to complete interviews during evenings and weekends
Nguyen et al., 2024	Recruitment Barriers and Potential Strategies for Inclusion of Older Asian Americans in Alzheimer's Disease Research	ADRD research			Narrative review	Asian Americans	Total k: Not reported Age: Not reported Sex: Not reported Cognitive Status: Not reported Country: U.S.	Passive recruitment methods, mistrust, language barrier, digital literacy, shame associated with AD diagnosis, lack of knowledge about AD, transportation		Establishing and maintaining community partnerships, community outreach events, bilingual personnel, social media for dissemination, key persons, remote recruitment and assessment with option for in-person for older participants, translation of recruitment/study materials		Not reported	Not reported
Olin et al., 2002	Increasing Ethnic Minority Participation in Alzheimer Disease Research	Focus on U.S.			Narrative review	Ethnic minorities	Total k: 8 Age: (51) Sex: Not reported Cognitive Status: Not reported Country: U.S.	Logistical barriers (transportation, need for stipends, financial costs)		Increasing level of personal contact with research staff, culturally competent / bilingual staff, pilot recruitment strategies, cross-validation of recruitment strategies		Not reported	Not reported
Savold et al., 2023	Barriers and solutions to Alzheimer’s disease clinical trial participation for Black Americans	AD clinical research recruitment			Systematic review	Black Americans	Total k: 26 Age: Not reported Sex: Not reported Cognitive Status: Mixed Country: U.S.	Mistrust, lack of knowledge/aware-ness on AD/research, cultural stigma dementia, financial barriers, transportation, time constraints		Building long-term sustainable community relationships, collaboration community organizations, study site at a trusted location, ethnically matched research staff, cultural competency training for researchers, cultural sensitive education materials, networking with community leaders/key persons, appropriate incentives (money, knowledge, transport), community-based participatory research methods, CAB		Not reported	Not reported
Shaw et al., 2022	Representation of Racial and Ethnic Minority Populations in Dementia Prevention Trials: A Systematic Review	ADRD prevention research			Systematic review	Ethnic minorities	Total k: 42 Age: 45+ (73 (ethnicity reported); 70 (ethnicity not reported)) Sex: 69 % (ethnicity reported); 64 % (ethnicity not reported) Cognitive Status: Healthy Country: U.S.	Lack of collecting and reporting ethno-racial information, mistrust, passive recruitment strategies (e.g. flyers), logistical barriers, eligibility criteria		Collaboration community organizations, incorporating technology to battle time burden, quota of ethics minority numbers		Not reported	Not reported
Villa-Castellar et al., 2022	A cultural approach to dementia — insights from US Latino and other minoritized groups	ADRD research recruitment			Perspective	Ethnic minorities	Total k: N/A Age: Not reported Sex: Not reported Cognitive Status: Mixed Country: U.S.	Cultural stigma dementia, perception of ageing/health, fear of invasiveness, seeing no relevance/benefit in study, mistrust, low health literacy levels (preventing from accessing healthcare)		Collaboration with community organizations, diversification research staff, social events (for dissemination), culturally/linguistically appropriate measurements, multilingual recruitment materials		Not reported	Culturally diversify research teams to reflect target communities.
Waheed et al., 2020	Recruitment and methodological issues in conducting dementia research in British ethnic minorities: A qualitative systematic review	ADRD research recruitment			Systematic review	Ethnic minorities	Total k: 33 Age: Not reported Sex: Not reported Cognitive Status: Mixed Country: UK	Cultural stigma dementia, collection/definition variable ethnicity, language barrier, logistical barriers (transportation, time burden, financial costs), low health literacy levels		Cultural competency training (researchers), ethnically matched research staff		Not reported	Reported difficulties with retention due to the “high mobility” (e.g., visiting home countries to fulfil obligations) of participants
Welsh et al., 1994	Issues Affecting Minority Participation in Research Studies of Alzheimer Disease	ADRD research recruitment			Narrative review	African Americans	Total k: N/A Age: Not reported Sex: Not reported Cognitive Status: Mixed Country: U.S.	Cultural stigma dementia, mistrust, logistical barriers (financial costs, transportation), lack of awareness, accessibility/stigma study site		Involving physicians in community, appropriate incentives (financial, or access to healthcare), transportation reimbursement, study site at a trusted location, at home visits, CAB, bilingual/bicultural research staff		Reports recruitment is better if personal contact is made with physicians, less appeal if 'for the sake of research'.	Not reported
Wong et al., 2019	Strategies for the Recruitment and Retention of Racial/Eth-nic Minorities in Alzheimer Disease and Dementia Clinical Research	ADRD clinical research recruitment			Systematic review	Ethnic minorities	Total k: 19 Age: Ranging from 64 to 77 Sex: ranging from 65 % to 76 % Cognitive Status: Mixed Country: U.S.	Not reported		Community outreach events (presentations, meetings, educational programming and materials), collaboration community leaders and organizations, face to face contact, collaboration health care providers (local physicians), referrals current participants, word-of-mouth recruitment, transportation provision		Collaboration with health care providers was most effective and advertisements were the least effective recruitment strategy.	Studies with the highest retention rates shared common retention strategies: follow-up communicat-ion through mail such as holiday cards and appointment reminders, maintaining community relationships through partnerships with local programs and hosting annual participant recognition events
Wrobel & Shapiro, 1999	Conducting Research with Urban Elders: Issues of Recruit-ment, Data Collection, and Home Visits	AD research recruitment			Narrative review	Ethnic minorities	Total k: N/A Age: Elderly Sex: Not reported Cognitive Status: Mixed Country: U.S.	Mistrust, logistical barriers (transportation, long wait, time constraints), lack of knowledge on AD, lack of education/aware-ness on research (benefits), lack of cultural compatible staff, language barrier		At home visits, short test measurements, flexible hours		Homes visits are reported as most effective	Loss of retention was caused by: failing health of participant, feelings of being misled during recruitment, not knowing the benefits or what is expected of them

Note: Age is reported as age range (mean age); Sex is reported as % female participants; cognitive status reported as either healthy or mixed meaning healthy and patient participants; AD = Alzheimer’s Disease; ADRD = Alzheimer’s Disease and Related Dementias; CAB = community advisory board; PI = principal investigator; UK = United Kingdom; U.S. = United States.

**Fig. 2:**
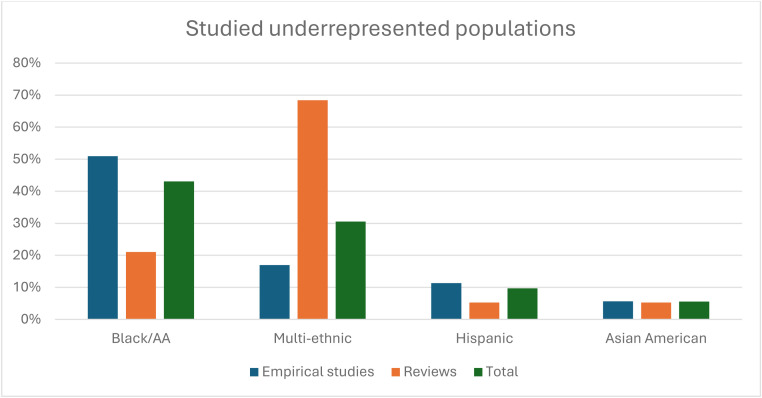
Distribution of studied ethnicities. Fig. 2: Distribution of studied underrepresented racial/ethnic groups Multi-ethnic includes cohorts from different ethnicities. Asian Americans include Chinese Americans, Korean Americans, Vietnamese Americans, and “Asian American” cohorts. Not shown in this graph are the American Indian and Alaska Native and British South Asian cohorts, each included in only one empirical study.

Over 90 % of the empirical studies and all but one review in this analysis were conducted in the U.S. One review [36] specifically focused on UK scientific literature. The majority of research focused on Black/African American (AA) participants (43.1 %), followed by multi-ethnic groups including cohorts with different ethnicities (30.6 %), Hispanic participants (9.7 %), and Asian-American participants (5.6 %) (Table 1, Fig. 2). The ‘multi-ethnic’ group generally includes cohorts from multiple underrepresented ethnic backgrounds, often including Black/AA, Hispanic, and Asian (Arab and Southeast Asian) cohorts.

Five empirical studies [[37], [38], [39], [40], [41]] (6.9 %) targeted the recruitment of older adults aged 55 and above in AD clinical trials. In these trials, more than 75 % of participants were White individuals. The researchers emphasized diversity across SES, age, and education within these cohorts. Among empirical studies reporting participant demographics, 56.5 % included a female majority with over 60 % female participants (Table 1). Only two studies [42,43] focused exclusively on male participants. None of the included studies reported beyond binary sex categories.

### 3.2 Barriers and facilitators to recruitment, engagement, and retention

**Fig. 3:**
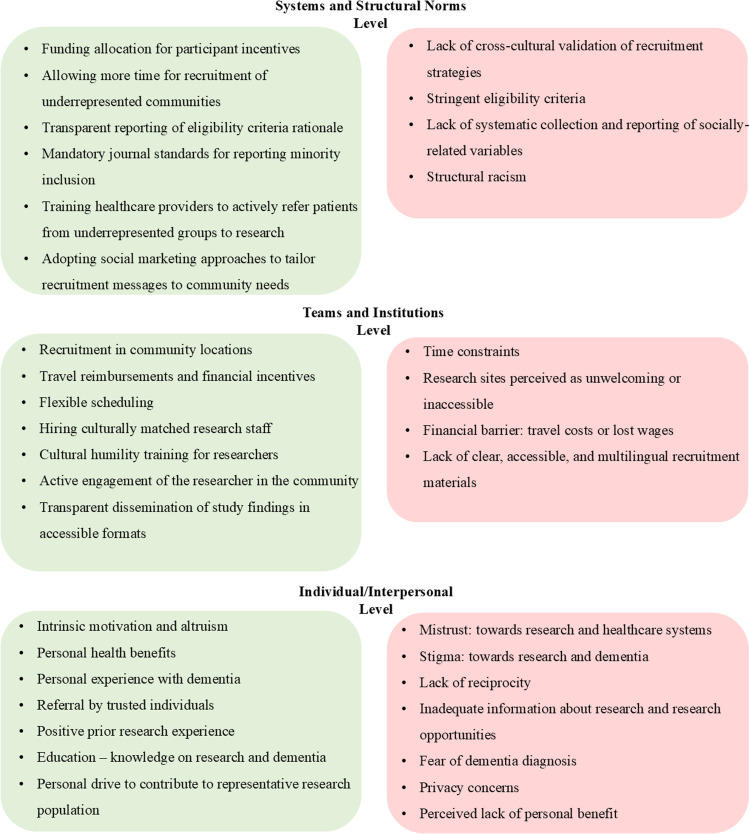
Overview of barriers and facilitators, categorized according to the conceptual structure described by Gilmore-Bykovskyi et al. [19].

For a general overview of the identified barriers and facilitators, see Fig. 3. In the following sections, the barriers and facilitators are shown in the context of five key statements.

**Statement 1: Mistrust is deeply rooted and serves as a significant barrier to research participation**.

Mistrust is one of the most frequently reported barriers to research participation at the Individual/Interpersonal level [24,26,27,38,42,[44], [45], [46], [47], [48], [49], [50], [51], [52], [53], [54], [55], [56], [57], [58], [59], [60], [61], [62], [63], [64], [65], [66], [67]]. This barrier is particularly emphasized in studies involving Black/AA populations [24,42,[44], [45], [46], [47], [48], [49], [50], [51], [52], [53], [54],^57^,^58^,^62^,^64^]. Mistrust can arise from a variety of factors, including past negative experiences or perceived risks, leading to hesitation in considering interventions as beneficial rather than harmful [24,27,44,63]. “Mistrust is often rooted in a historical context of unfulfilled promises and deliberate harmful treatment or abuse of minoritized populations,” as stated in a review by Ballard et al. [57]. Significant examples of medical injustice in the historical context include the Tuskegee Syphilis Study (where AA men were denied treatment for syphilis), and the case of Henrietta Lacks (whose cervical cancer cells were taken and are still used in research without her consent) [28]. These events have contributed to participants believing they will be tested on and treated as “guinea pigs” [44,49,51].

Skepticism towards research is exacerbated by the lack of reparations or redress by government authorities [57]. Additionally, there is a fear of “being treated unfairly, and therefore only the possibility to receive a placebo and not the actual medicine” in empirical studies [44,45,47,51,68]. Lastly, another effect of mistrust is seen in regard to privacy concerns, as the safekeeping of personal data was found to be questioned by participants [44,50,52,53,56,61,69].

### 3.3 Facilitators against mistrust

One of the most cited facilitators for research engagement at the Individual/Interpersonal level is participants' intrinsic motivation or altruism [27,41,42,44,47,48,50,53,57,61,[69], [70], [71], [72], [73], [74], [75]]. Many participants are driven by the idea of contributing to a cure [44], or by creating a more diverse research landscape [45,50,70,76]. A personal connection to dementia, whether through experiences with family members or friends or personal stories shared by researchers, enhances relatability and trust, as evidenced in both empirical studies and reviews [24,42,48,50,73,76]. Personal health benefits, such as gaining health insights or research aligning with personal interest [38,41,42,47,53,61,[69], [70], [71],^73^,^74^,^77^], also motivate participation.

Referrals by current participants [28,33,61,75,76,78] and trusted professionals, like physicians, may serve as powerful motivators, especially given the role trusted figures command in communities [33,47,79]. Involving referring physicians in the recruitment stage has been suggested as an effective engagement strategy by one review and one empirical study [64,79]. Additionally, participants with past positive research experiences are more likely to participate again, underscoring the impact of (positive) prior engagement [47,73,74].

These facilitators underscore the importance of trust, personal relevance, and positive prior experiences in motivating research participation.

**Statement 2: Stigma and limited research literacy perpetuate fear and misconceptions, undermining participation in research.**

#### 3.3.1 Research stigma and limited research literacy

Inadequate knowledge or information about research [27,36,46,47,52,61,62,69,70,75,[80], [81], [82]] may result in stigma surrounding research participation. Stigma is compounded by concerns about the potential side effects of study medications [24,27,44,60,69], receiving a placebo [44,45,47,51,68], and the invasiveness of procedures [27,53,54,63,68]. These concerns were specifically reported in studies conducted within brain health registries, where participants enroll with the possibility of being invited to studies that may involve the collection of cerebrospinal fluid or blood samples. Additionally, limited awareness of research opportunities [24,44,52,55,64,71,81,82], and the perceived lack of personal benefits [24,26,27,44,49,50,52,63,70,81] further hinder participation.

#### 3.3.2 Dementia stigma and health knowledge

Stigma surrounding dementia presents a significant barrier to participation in dementia research among underrepresented populations [24,36,44,56,59,62,63,66,67,75,82]. Inadequate knowledge of dementia and health literacy [36,50,55,61,63,[65], [66], [67],[80], [81], [82], [83]] may result in misconceptions, which heighten stigma. This misinformation, combined with cultural differences in how dementia is perceived—for example, as a disease versus a natural part of aging—can discourage individuals from engaging in dementia prevention research [59,60,63,81].

This stigma can also extend to family members. In a study by Hinton et al. [84]., the reasons individuals provided for declining participation could be traced back to objections from their adult children, who expressed concerns about the implications of a possible diagnosis. In addition to these familial concerns, participants themselves reported fear of diagnosis—including “the fear of being confronted with cognitive decline”—as a significant deterrent to engaging in research involving memory testing [66,67,69,75,84].

Interestingly, Amofa et al. [44]. report that participants (Black American adults residing in North Florida) express greater willingness to engage in early preventive measures to delay or prevent cognitive disorders rather than in clinical studies conducted after cognitive decline begins. As they note: “Although stigma and shame were reported as potential barriers to participation in memory disorder research during the focus group, our participants disagreed that this posed a barrier to dementia screening. This aligns with participants’ expression of interest in early measures to prevent or delay the onset of cognitive disorders like dementia, as opposed to identification after the fact, which carries more stigma (speaking to concerns surrounding confidentiality).”

### 3.4 Facilitators to combat stigma and limited research literacy

Increasing research literacy helps reduce misconceptions and uncertainties around intervention studies and could make individuals more willing to participate [24,60]. Education on both research procedures [24,28,33,45,47,51,60,62,67,75,80,83,[85], [86], [87]], and specific conditions such as AD [28,45,51,58,66,76,83,85,88,89] plays a crucial role in combating stigma and improving research literacy. Education can be delivered through various formats, with studies showing that different approaches can be effective. For example, Fritsch et al. [85] utilized an educational play to engage individuals both emotionally and cognitively, helping AA participants better understand AD and its implications. Similarly, Sewell et al. [86] reported on the effect of multilingual educational videos that provided clear and accessible information about dementia research, ultimately increasing research volunteerism, trust, and personal safety among elderly Hispanic participants. Bardach et al. [45] reported that “participants believed instilling a sense of responsibility for brain health from a young age could lay the foundation for future research involvement.”

These examples highlight how accessible and culturally sensitive education can play a key role in creating trust and increasing willingness to participate in research.

**Statement 3: Logistical and financial constraints create structural barriers that restrict research participation and widen disparities.**

Logistical barriers play a significant role in hindering research participation and engagement among underrepresented populations at the Teams and Institutions level. Time constraints [24,27,36,39,42,44,48,53,54,[56], [57], [58], [59],^61^,^62^,^65^,^66^,^70^,^71^,^74^,^82^,^90^,^91^] often limit participation, particularly when study designs impose rigid schedules, weekday-only sessions, or lengthy durations that conflict with work and family responsibilities [44,54]. Furthermore, transportation issues to the study site present a substantial challenge [24,27,36,39,44,[53], [54], [55],^58^,^59^,^62^,[64], [65], [66], [67],^69^,^71^,^81^,^83^,^92^]. The stigma associated with research sites situated in affluent neighborhoods [80,82], and the accessibility of these locations can discourage participation. Moreover, disadvantaged neighborhoods are underrepresented in ADRD recruitment registries, which limits socioeconomic diversity and further reinforces disparities in access to research opportunities [40]. This underrepresentation is closely linked to financial barriers, as potential participants may lack the resources to cover associated costs, such as travel expenses or lost wages due to time away from work [24,36,43,48,55,56,[60], [61], [62],^64^,^80^,^83^,^92^].

### 3.5 Facilitators to overcome logistical barriers

To address logistical barriers, various strategies have been identified at the Team and Institutional levels. Holding recruitment sessions in familiar, community-based locations and offering in-home visits [24,[26], [27], [28],^39^,[54], [55], [56],^60^,^62^,^64^,^65^,^67^,^72^,^75^,^82^,^84^,^93^] has been suggested as effective for “enhancing accessibility and comfort, especially among those with limited transportation options.” Additionally, mapping the target population using Geographic Information Systems (GIS) could help identify areas with high concentrations of potential participants, enabling more targeted recruitment [94]. Rotating research locations to reach different neighborhoods has also been suggested to diversify participant pools and make studies more geographically inclusive [24,95].

Additionally, providing travel reimbursements or a taxi-service [24,27,39,47,51,[53], [54], [55],^59^,^64^,^66^,^73^,^75^,^80^,^84^] alleviates financial strain, enabling participation without out-of-pocket expenses. Practical incentives, including monetary incentives [24,27,39,42,47,48,51,[53], [54], [55],^60^,^62^,^64^,^68^,^73^,^75^,^76^,^96^], or access to healthcare [64,75], help to acknowledge the time and effort required for participation. One study [96] found no difference in participation rates when increasing compensation from $50 to $100, suggesting remuneration may serve more as a gesture of recognition than a financial incentive. Another study [39] reported no significant differences in compensation preferences by “race”, though individuals without personal familiarity with AD were more likely to prefer financial incentives. Lastly, flexible research participation times [24,54,60,65,66,75] were noted to support engagement among individuals with complex work and family responsibilities.

Together, specific logistical strategies, such as holding recruitment sessions in familiar and trusted locations, can help lower practical barriers to participation, making research more accessible to individuals with varying needs and responsibilities.

**Statement 4: Inadequate and impersonal communication, and a lack of diversity within research teams weaken trust and hinder research engagement**

#### 3.5.1 Recruitment materials

Effective research communication plays a critical role in participants' research understanding and engagement. Reviews by Gilmore-Bykovskyi et al. [27] and Godbole et al. [24] highlight that insufficient information about study procedures is a significant barrier to participation. This communication gap can sometimes be attributed to participants’ varying levels of English proficiency and the lack of study/recruitment materials in different languages [24,36,55,65,[81], [82], [83],^95^,^97^]. Furthermore, issues with literal translations can exacerbate this barrier when translating materials to other languages. For instance, Ajrouch et al. [95] report that the literal translation of the word "dementia" into Arabic as "Kahraf" carries derogatory and offensive connotations, alienating potential participants.

#### 3.5.2 Lack of diversity in the research team

A lack of representation and diversity within the research staff itself can contribute to mistrust and disconnection with potential participants [60,61,65,82]. Furthermore, a ‘one size fits all’ approach to recruitment was found to be problematic, as it ‘failed to acknowledge the within-group diversity of participants [57,91,95].’

#### 3.5.3 Dissemination of findings

Scharff et al. [52] report that many participants attributed their confusion about research to inadequate public dissemination of information. Even when findings are shared, they are “often not communicated clearly, in an accessible way, and understandably, limiting their usefulness for participants.” In an interview study with four church leaders by Bonner et al. [46], a paternalistic attitude among researchers was identified as a barrier. The church leaders highlighted the lack of visibility of the principal investigator (PI) and a lack of collaboration in interpreting the study findings, resulting in feelings of ‘helicopter research,’ where researchers gather data and leave without providing feedback. This sentiment was not isolated, similar concerns about researchers failing to reciprocate or follow up meaningfully were echoed across additional studies, appearing explicitly in one review [57] and two empirical studies [28,44].

### 3.6 Facilitators to strengthen trust through inclusive communication and diverse, community-engaged research practices

#### 3.6.1 Community involvement in recruitment

Addressing relational and cultural barriers requires a proactive and inclusive approach that builds trust and engagement across diverse communities. One effective strategy is to directly involve community members in the recruitment process. Establishing community advisory boards (CABs) composed of members from the target population [57,59,62,64,66,76,79,86,88,98] could further inform researchers on a culturally responsive approach. Additionally, employing culturally or ethnically matched staff has been widely recognized as a facilitator [24,26,27,36,47,49,56,58,60,62,64,66,67,75,77,83,84,92,99], as participants often feel more comfortable engaging with researchers who reflect their own cultural backgrounds. Providing cultural humility training for researchers [36,61,62,82] can further enhance cultural competence and create more inclusive research environments.

Forming partnerships or endorsements with trusted community organizations, such as churches and local institutions, has also been suggested to effectively build trust and encourage research participation [24,26,33,45,[55], [56], [57],^60^,^62^,^63^,^66^,^67^,^75^,^78^,^79^,^87^,^98^,^99^]. Engaging key community figures as liaisons between researchers and participants can bridge trust gaps [24,33,46,56,[58], [59], [60],^62^,^66^,^67^,^82^,^86^,^91^,^95^]. Moreover, individuals from the community can be trained as lay educators to share research information [86]. This community-driven approach can also be extended to involve family members as connectors in recruitment by applying snowball sampling strategies [28,78,100].

#### 3.6.2 Building trust through sustained community presence

Maintaining a visible and active presence in the community is another critical facilitator. Principal investigators (PIs) and research teams who engage with the community beyond the study context [44,46,49,51,58,59,66,67,75,101] “would signal altruism and can create a space that makes it safe to ask questions [75].” Organizing community outreach events, such as health fairs or food gatherings [28,33,56,57,59,63,66,67,71,78,90,93,95,98], provides informal opportunities for face-to-face interactions that can further build mutual trust. Similarly, hosting open discussions and small group information sessions to address concerns, mistrust, and preferences [28,33,[45], [46], [47],^52^,^59^,^60^,^72^,^77^,^79^,^86^,^87^] can help create transparency and encourage participation. During these sessions, it is essential to have transparent communication about the research goals, benefits, and procedures to manage expectations and build trust [24,27,45,46,51,57,58,75].

#### 3.6.3 Sharing findings and sustaining community trust

Meaningful engagement extends beyond data collection to how findings are shared. Disseminating results at the original intervention site [49] and providing accessible, multilingual summaries through mail or social media ensures that participants feel valued and informed [45,52,75,76]. Delivering interventions in group settings [43,44,83] can also reduce participation barriers by creating a sense of community within the research process. Ongoing community involvement after study completion can help in building long-term bidirectional relationships, reinforcing trust, and laying the groundwork for future collaborations [28,45,58,62,66,67,98].

Together, these strategies demonstrate that culturally responsive communication, sustained across the research process and beyond, can build trust and support long-term engagement with underrepresented communities.

**Statement 5: Systemic and policy-level shortcomings reinforce exclusion and limit the inclusivity of research efforts.**

At the Systems and Structural Norms level, systemic barriers hinder the participation of underrepresented populations in research. One major challenge is the lack of effective centralized recruitment efforts [93] and cross-cultural validation of recruitment strategies [92]. Raman et al. [93]. report how prescreening databases (used to assess participant eligibility prior to informed consent) could support real-time evaluation of outreach and screening efforts. However, in the absence of centralized coordination and shared best practices, the potential of these tools to identify when and where specific groups are lost in the recruitment process remains underutilized. As a result, research teams operate in isolation, limiting broader engagement.

Stringent eligibility criteria also act as a barrier, often excluding participants from diverse backgrounds due to narrowly defined inclusion parameters [26,60,61,81,82,90,93]. Additionally, there is a lack of systematic collection and reporting of ethnicity-related variables, making it difficult to assess participation equity and tailor recruitment strategies to underrepresented populations [24,36]. While this is often seen at the Team and Institutional level, it may also reflect broader policy gaps, as journals and funding bodies do not consistently require detailed reporting of eligibility criteria or participant demographics.

### 3.7 Facilitators for overcoming policy and systemic barriers

To address these systemic challenges, several policy-level strategies have been identified to enhance research inclusivity. Allocating funding for appropriate participant incentives [82,101] ensures that financial barriers, such as travel costs or time off from work, are alleviated. Furthermore, building more time into the recruitment process acknowledges the additional effort required to engage underrepresented communities meaningfully [28,59,81].

Transparent reporting of the rationale behind eligibility criteria promotes accountability and encourages inclusive study designs [26,60]. Additionally, implementing publication standards, such as requiring journals to set quotas for reporting the inclusion of ethnic minority participants, can be an incentive for researchers to prioritize diversity in their studies [17,82].

Institutionalizing physician referral training at the policy-level ensures that healthcare providers are equipped to actively refer diverse patients to research opportunities [79]. By embedding this training into healthcare systems and institutional policies, it creates a standardized approach that broadens outreach beyond individual research teams. This systemic strategy could enhance the role of trusted medical professionals in recruitment efforts [79].

Adopting alternative recruitment strategies, such as a social marketing approach, involves conducting a marketing analysis to understand community needs and preferences before designing recruitment campaigns. This targeted approach ensures that messaging resonates with specific populations, improving engagement and participation [79]. Walker et al. emphasize tailoring recruitment strategies to the target population’s characteristics and history [28]. At the policy-level, it is essential to promote community involvement throughout the full research cycle, ensuring that recruitment is culturally responsive and supported by institutional policies and frameworks that prioritize inclusivity and diversity [82,100].

Collectively, these policy and system level strategies can dismantle structural barriers and promote a more inclusive research landscape.

### 3.8 Comparing recruitment initiatives

Nineteen empirical studies and six reviews discussed the effectiveness of recruitment. Similar to the systematic review of Gilmore-Bykovskyi et al. [27], our study also found that most studies evaluated recruitment either by tracking the number of new enrollments for one method compared to another, e.g., more participants recruited from those attending community events vs. those who do not attend, or by comparing recruitment numbers of targeted communities to previous years [28,37,40,55,86,90,91,97,98,100]. For instance, Graham et al. [78] report on the effectiveness of health fairs and tailored advertisements in recruiting Black Americans. Black male participants, specifically, were mostly recruited through family referrals, “indicating a need for trust in their decision to participate in clinical trials.”

For articles that compared methods, recruitment via community outreach initiatives or trusted community members tended to be more effective than recruitment via primary health care referrals, dissemination of brochures or flyers, or direct mail campaigns [28,47,55,77,80,91,98]. Additionally, Gabel et al. [96] report on the positive effects of remuneration in the recruitment across all participants, regardless of ethnicity or income.

While many studies identified barriers and facilitators to recruitment, few explicitly used validated models or frameworks to guide these efforts. Notable exceptions include studies using the Socio-ecological model [81], the Minority Recruitment Model [100], and the Transtheoretical behavior change model [88,89].

### 3.9 Retention of a diverse study population

Eighteen empirical studies and 11 reviews reported on participant retention. Other studies either did not address retention explicitly, suggested it as a future research focus, or mentioned “recruitment and retention” in their introductions and discussions, yet only reported recruitment strategies in the results [28,55,80,81,87,90]. This issue was highlighted in Gilmore-Bykovskyi et al.'s [27] systematic review, which noted that studies often failed to distinguish whether specific efforts targeted recruitment or retention (p. 760). Indeed, Wong et al. [33] report the paucity of evidence on the effectiveness of retention strategies in ethnic minority populations, noting a lack of data on strategies targeting Hispanic, Asian American, and Native American/American Indian populations.

For articles which addressed retention, the following was highlighted as effective: (a) maintaining a connection with relevant communities throughout the research process and its community leaders or trusted members, (b) maintaining a relationship with the participant as well as their life companions, e.g. spouse, child, friend; and, (c) follow-through with what is promised during recruitment, e.g. dissemination of results from tests or feedback [41,95,98].

## 4 Discussion

This scoping review mapped barriers and facilitators to the recruitment, engagement, and retention of REM populations in dementia prevention research. Synthesizing findings from both empirical studies and reviews, and structuring them according to the conceptual structure described by Gilmore-Bykovskyi’ et al. [21], the review identified five thematic statements spanning Individual/Interpersonal, Teams and Institutions, and Systems and Structural Norms levels. Across all themes, a recurring emphasis emerged on the importance of trust, community engagement, and responsiveness to the sociocultural context in shaping successful inclusion strategies. Mistrust toward scientific research, rooted in both historical abuse and ongoing structural inequities, was the most frequently reported barrier (Statement 1). In the context of dementia prevention, mistrust may be heightened, as participants are often cognitively healthy. Moreover, the benefits of participation are not immediately apparent, which can lead to reluctance to join studies [58].

Research and dementia-related stigma (Statement 2), combined with limited familiarity with research, seem to further reduce willingness to engage with dementia prevention initiatives. Next, structural and logistical barriers (Statement 3), such as complex study procedures, exclusionary eligibility criteria, and burdensome trial logistics, limit access to research. Communication gaps, lack of cultural concordance between research teams and participants, and minimal transparency during and after the study (Statement 4) also hampered engagement and trust. Finally, on the Systems and Structural Norms level (Statement 5), the absence of inclusive trial infrastructure and policy-level accountability mechanisms perpetuated the exclusion of REM populations from dementia prevention science.

These findings align with barriers identified in other fields, such as cardiovascular [102] and cancer [103] research, where mistrust, lack of diversity among research teams, language, and logistical access were reported to obstruct inclusion in a similar manner [104]. However, dementia prevention poses distinct challenges. While similar challenges exist in other areas of prevention, such as cardiovascular disease (e.g., long trial durations, demanding interventions, and asymptomatic participants), dementia carries a unique stigma. This stigma can contribute to the perception that prevention is not urgent, especially when individuals are asymptomatic or do not recognize dementia as a valid health condition. For instance, some cultures may view dementia as part of ‘normal aging’ or attribute it to fatalism [105,106], which may amplify hesitancy and attrition. These nuances underscore the need for field-specific strategies that balance ethical transparency with culturally appropriate framing of risk and benefit [18].

The majority of the included studies were conducted in the U.S., with a predominant focus on Black or AA communities. While this aligns with established patterns of health disparities in the U.S. context, there remains limited evidence on recruitment, engagement, and retention strategies for other REM populations, particularly in Europe or low- and middle-income countries (LMICs) worldwide. This U.S.-centric focus leaves a knowledge gap on how strategies might translate or require adaptation in regions with different healthcare systems, migration histories, and sociocultural dynamics. Underrepresented populations differ across countries, shaped by distinct historical, social, and political contexts such as colonial histories, labor migration, or refugee movements [28]. These dynamics affect trust, communication preferences, and social and structural barriers to engagement, indicating the need for recruitment strategies tailored to country-specific demographic and cultural realities.

Similarly, populations in LMICs face distinct challenges, such as limited healthcare access and less highly developed research and clinical trial infrastructure [107,108], which may alter the barriers and facilitators identified in U.S.-based studies. Sociocultural and economic dynamics, including differing healthcare systems, migration patterns, and public health priorities, further shape both participation in research and intervention outcomes [109,110]. Moreover, cultural perceptions of dementia, such as interpretations of symptoms as a result of normal aging rather than a disease, and stigma surrounding it, alongside low public awareness, further reduce participation in research and early prevention efforts [14,108]. Socioeconomic vulnerabilities, such as low educational attainment, insufficiently trained healthcare providers, inadequate health policy support, and constrained financial resources [108,109] create a distinct set of obstacles that may not be fully captured by barriers identified in predominantly U.S.-based studies. These contextual differences indicate the need to develop context specific strategies tailored to the sociocultural, economic, and healthcare realities of diverse global populations. The scarcity of dementia prevention trials in LMICs emphasizes the necessity of both local and multinational studies that can explore these challenges across diverse populations, ensuring that globally applicable strategies are developed to prevent dementia [109,110].

Some of the reported barriers and facilitators are not unique to the U.S. context. A dementia prevention study conducted in Peru highlighted the importance of culturally grounded approaches, including using linguistically appropriate materials and diverse research teams to overcome communication barriers and to build trust. The authors also emphasized that first in-person contact, rather than digital outreach, was critical for participant engagement, aligning with cultural values of ‘*personalismo’*, or warm, interpersonal connections [97]. However, other studies (Table 1) have shown that digital recruitment strategies, including social media platforms like Facebook, have been successfully employed to engage underrepresented populations [111]. These platforms offer the opportunity for dissemination [67], and increased engagement via the opportunity for discussion [76], as such extending the reach of traditional recruitment methods. Such findings reinforce the need for inclusion strategies to be locally responsive and embedded in relevant cultural norms. While digital methods may be effective in some contexts, they could also introduce challenges related to digital literacy and access [111], which must be carefully considered when designing recruitment strategies.

A notable sex imbalance was observed in the demographics, where 56.6 % of the included empirical studies had female participants. Only two studies [42,43] focused exclusively on male participants, highlighting a significant knowledge gap regarding sex-specific barriers. Beyond sex, age-related challenges and their intersection with other factors (e.g., ethnicity, SES) remain insufficiently explored. The interplay of these factors creates syndemic risks [14], where multiple co-occurring disadvantages like racism, caregiving roles, and SES influence barriers to participation. Education, however, may partly buffer against these challenges. Higher educational attainment is frequently associated with improved health literacy [112], enhanced access to healthcare through socioeconomic advantage [113], and has been linked to higher participation in health research [89,114]. On the other hand, lower educational attainment may pose additional barriers, such as difficulty meeting eligibility criteria or challenges in understanding informed consent procedures, which can hinder participation in research [26]. Moreover, knowledge on dementia and higher research literacy was highlighted as an important facilitator in several included studies (Table 1), as informed participants are more likely to engage in research. Applying an intersectional lens is essential to understanding how these overlapping factors shape participation barriers and engagement strategies [12,13]. For instance, the HANDLS study [115] demonstrated that effective recruitment and retention required tailoring strategies to ethnicity, SES, sex, and age. This illustrates how syndemic risks must be considered to build trust and sustain participation among diverse older populations.

Although recruitment received substantial attention, retention strategies were rarely discussed and examined. While recruitment strategies may also be effective for participant retention, this cannot be assumed and therefore should be evaluated and considered separately. Similarly, multiple reviews on recruitment in dementia clinical trials emphasized the lack of clarity in studies regarding whether their strategies addressed recruitment or retention, and urged future research to consider this limitation [18,92,116]. Given the prolonged nature of dementia prevention trials, this omission is concerning. This gap may be particularly significant for older adults who face intersecting challenges, such as caregiving responsibilities, health comorbidities, or digital literacy barriers, that affect long-term participation. Hence, sustained participation requires more than initial interest. It demands ongoing communication, transparency, and prolonged community partnership. Participants are more likely to remain in a study or research initiative if they, their loved ones, or community leaders feel that researchers are genuinely committed to their well-being. This trust is built not only through hosting community events but also by establishing a trusted relationship with researchers through their continuous commitment to the community's well-being and appreciation of participants’ involvement [28,95]. Without targeted efforts to support long-term engagement, the risk of attrition may undermine trial validity and equity.

Few studies rigorously evaluated the effectiveness of recruitment strategies, often describing community-based or culturally tailored approaches that lack standardized comparative metrics. A key limitation is the absence of consistent measures, such as recruitment rates, screen-failure rates, or costs per enrolled participant, which would enable comparison across studies. Moreover, the limited use of validated models or frameworks suggests a need for both structured, evidence-based approaches to recruitment strategies, which could enhance the consistency and effectiveness. Additionally, some studies did not consistently report key demographics, including sex, age, and participant numbers, which are essential for assessing the representativeness of study populations. Future research should prioritize consistent reporting of these metrics to improve inclusivity and generalizability. Treweek et al. [117] highlight that recruitment for interventions is rarely tested in robust, controlled ways, leaving uncertainty about which strategies truly improve participation. Similarly, Raman et al. [93] emphasize the need for better reporting of participant flow and representativeness metrics in ADRD research to assess inclusivity. While this review focuses on prevention studies and research participation, we acknowledge that epidemiological studies, particularly those focused on risk factors and risk prediction, could provide additional insights. Future studies on barriers and facilitators in these areas could complement and build on our findings. Moreover, future studies should adopt standardized methodological approaches to evaluate recruitment and retention strategies, including clear reporting on metrics such as recruitment and retention rates, costs per participant, and ethnic diversity in the study population. Without consistent evaluation and reporting methods, promising approaches cannot be optimized or scaled across diverse research settings.

Efforts to improve REM representation in dementia prevention research must go beyond individual level strategies and address broader institutional and policy reforms. This includes adopting inclusive recruitment metrics, embedding community partnerships in research funding models, and supporting culturally grounded co-design approaches. Researchers should consider intersectionality, including ethnicity, gender, SES, and other social determinants [12]. Additionally, further research is needed on country-specific population barriers. Building on these implications, Box 1 summarizes key practice and policy recommendations derived from the five thematic statements.

**Box 1:** Practice and policy recommendations.

Practice & Policy Recommendations
1. **Build trust through sustained community engagement:** Long-term, transparent partnerships with community organizations and trusted leaders are essential to address mistrust and historical marginalization. Researchers should establish community advisory boards and involve community representatives early in the study design process.
2. **Improve research literacy and reduce stigma through culturally tailored outreach:** Provide accessible educational materials about dementia prevention and research participation in culturally and linguistically appropriate formats. Emphasize the value and safety of prevention studies to reduce stigma and misconceptions.
3. **Reduce logistical and financial barriers through targeted population planning**: Conduct a target population analysis to understand community-specific needs, preferences, and potential barriers. Use this knowledge to allocate resources effectively, e.g., by offering transportation, childcare, flexible scheduling, and fair compensation to enable participation among individuals facing socioeconomic or caregiving constraints.
4. **Strengthen communication and diversify research teams:** Enhance transparency and cultural responsiveness in communication by ensuring research teams reflect the diversity of the populations they serve. Employ bilingual or bicultural staff, maintain consistent communication throughout the study, and create a culturally competent environment that supports participant retention.
5. **Improve reporting of ethnoracial demographics**: Future research should prioritize transparent consistent reporting of ethnocracial demographics in study participants. Clear demographic reporting allows for better comparison of findings across studies and ensures inclusivity in research.

Box 1: Practice and policy recommendations.

The numbered recommendations in this box correspond to the thematic statements outlined in the results section (Statements 1 – 5). These actionable points summarize key strategies for improving recruitment, engagement, and retention of underrepresented populations.

This study has a few limitations. As this was a scoping review, no formal appraisal of methodological quality was conducted, and studies were included regardless of their quality. This approach was necessary to capture the breadth of available evidence. A uniform quality assessment would have been challenging given the mix of reviews and empirical studies. Most included studies had small, single-country samples, which limited their generalizability. Finally, the inclusion of both literature reviews and empirical studies could have introduced heterogeneity, although this was mitigated by reporting their numbers separately to clarify the source of evidence.

This review provides a structured synthesis of the literature on REM participation in dementia prevention studies. By applying a multi-level conceptual structure and integrating diverse sources, it highlights the most pressing challenges, promising strategies, and knowledge gaps across countries and populations. These findings can guide future trial design, institutional practices, and policy agendas to make dementia prevention research more inclusive and equitable. Overall, the review highlights the importance of tailored, multi-level strategies to ensure equitable participation and sustained engagement of REM populations in dementia prevention research. It emphasizes the need for context-specific approaches and systemic reforms to enhance inclusivity across diverse settings. However, the effectiveness of recruitment strategies is rarely tested in robust, controlled ways, leaving uncertainty about which strategies truly improve participation [117]. To overcome this limitation, future research should prioritize more structured approaches that rigorously evaluate and compare recruitment methods to optimize strategies for engaging REM populations.

## 5 Conclusion

This review highlights that barriers to participation in dementia prevention research among underrepresented groups are multifaceted and require tailored, context-sensitive strategies. Building trust through sustained community partnerships and culturally aligned approaches is essential. Yet, while inclusive recruitment has received increasing attention, retention remains underexplored despite its importance for long-term dementia prevention trials. Only through transparent reporting of recruitment practices and eligibility criteria can dementia prevention research become comparable across contexts and genuinely inclusive.

## Funding

10.13039/501100001826ZonMw (10510032120004, NDPI consortium)

## Conflicts of interest

AFR has received support to attend conferences from Alzheimer Nederland. SF has received consulting fees from Biogen (paid to her organization). Research of NCV has been funded by ZonMW, Alzheimer Nederland, Health∼Holland, Topsector Life Sciences & Health, EISAI and Amsterdam public health research institute.

## Declaration of generative AI and AI-assisted technologies in the manuscript preparation process

During the preparation of this work the author(s) used ChatGPT 5.1 in order to proofread the manuscript. After using this tool/service, the author(s) reviewed and edited the content as needed and take(s) full responsibility for the content of the published article.

## Data statement

Search terms and included medical libraries can be found in Appendix/Supplement 1. All the included articles of this review, and the reported barriers/facilitators are available in Table 1.

## Ethical statement

As this is a scoping review, no informed consent or ethical approval was required. Additionally, as scoping reviews typically do not require protocol registration, our protocol was not registered in PROSPERO or Open Science Framework (OSF).

## CRediT authorship contribution statement

**A.F. Rirash:** Writing – review & editing, Writing – original draft, Visualization, Validation, Project administration, Methodology, Investigation, Formal analysis, Data curation, Conceptualization. **S. Franzen:** Writing – review & editing, Writing – original draft, Supervision, Funding acquisition, Conceptualization. **R. Bourdage:** Writing – original draft, Visualization, Investigation, Formal analysis, Data curation. **E. Kreuk:** Formal analysis. **N.C. Visser:** Writing – review & editing, Conceptualization. **G.M. Babulal:** Writing – review & editing. **E. van den Berg:** Writing – review & editing, Writing – original draft, Validation, Supervision, Conceptualization. **J.M. Papma:** Writing – review & editing, Writing – original draft, Validation, Supervision, Methodology, Conceptualization, Funding acquisition.

## Declaration of competing interest

The authors declare the following financial interests/personal relationships which may be considered as potential competing interests:

A. F. Rirash reports a relationship with Alzheimer Netherlands that includes: travel reimbursement. S. Franzen reports a relationship with Biogen Inc that includes: consulting or advisory. N. C. Visser reports a relationship with Health Holland that includes: funding grants. N. C. Visser reports a relationship with Topsector Life Sciences & Health that includes: funding grants. N. C. Visser reports a relationship with Eisai Inc that includes: funding grants. N. C. Visser reports a relationship with Amsterdam Public Health Research Institute that includes: funding grants. N. C. Visser reports a relationship with Alzheimer Netherlands that includes: funding grants. If there are other authors, they declare that they have no known competing financial interests or personal relationships that could have appeared to influence the work reported in this paper.

## Appendix Supplementary materials

Image, application 1
