# Supplementary material for: Barriers and facilitators to recruitment, engagement, and retention of underrepresented populations in dementia prevention research: a scoping review
Source: J Prev Alzheimers Dis. 2026 Apr 4;13(6):100557. doi: 10.1016/j.tjpad.2026.100557 (PMC13089022; doi:10.1016/j.tjpad.2026.100557)
Supplement: Supplementary file 1 [file mmc1.docx]

**Appendix 1**

**Search strategy**

| **Database searched** | **Platform** | **Years of coverage** | **Records** | **Records after duplicates removed** |
| --- | --- | --- | --- | --- |
| Medline ALL | Ovid | 1946 – July 1 2025 | 1011 | 997 |
| Embase | Embase.com | 1971 – July 1 2025 | 1775 | 1041 |
| Web of Science Core Collection* | Web of Knowledge | 1975 – July 1 2025 | 1196 | 431 |
| CINAHL** | EBSCO | 1982 – July 1 2025 | 616 | 205 |
| Global Index Medicus*** | globalindexmedicus.net |  | 319 | 307 |
| **Total** | | | **4917** | **2981** |

*Science Citation Index Expanded (1975-present) ; Social Sciences Citation Index (1975-present) ; Arts & Humanities Citation Index (1975-present) ; Conference Proceedings Citation Index- Science (1990-present) ; Conference Proceedings Citation Index- Social Science & Humanities (1990-present) ; Emerging Sources Citation Index (2005-present)
**Limited to Academic Journals

*** Includes African Index Medicus (AIM), Index Medicus for the Eastern Mediterranean Region (IMEMR), Index Medicus for the South-East Asia Region (IMSEAR), Latin America and the Caribbean Literature on Health Sciences (LILACS), Western Pacific Region Index Medicus (WPRO)
No other database limits were used than those specified in the search strategies

**Medline**

("Minority Groups"/ OR "Ethnic and Racial Minorities"/ OR "Minority Health"/ OR "Refugees"/ OR "Transients and Migrants"/ OR (((underrepresent* OR disadvant*) ADJ3 (group* OR people* OR person OR persons OR patient* OR elder* OR client* OR adult*)) OR minorit* OR refugee* OR immigrant* OR migrant* OR hard-to-reach* OR ethnic* OR racial* OR race* OR ((social*) ADJ3 (disadvant*))).ab,ti,kf.) **AND** ("Evaluation Study".pt. OR exp "Evaluation Studies as Topic"/ OR (barrier* OR challenge* OR facilitator* OR obstacle*).ab,ti,kf. OR (evaluat* OR difficult* OR enable* OR promot*).ti.) **AND** ("Dementia"/ OR exp "Alzheimer Disease"/ OR (dement* OR Alzheimer*).ab,ti,kf.)

**Embase**

('underrepresented minority'/exp OR 'ethnic or racial aspects'/exp OR 'minority group'/exp OR 'minority health'/exp OR 'refugee'/de OR 'migrant'/exp OR 'ancestry group'/exp/mj OR (((underrepresent* OR disadvant*) NEAR/3 (group* OR people* OR person OR persons OR patient* OR elder* OR client* OR adult*)) OR minorit* OR refugee* OR immigrant* OR migrant* OR hard-to-reach* OR ethnic* OR racial* OR race* OR ((social*) NEAR/3 (disadvant*))):ab,ti,kw) **AND** ('barriers'/exp OR 'facilitator'/exp OR 'evaluation study'/exp OR (barrier* OR challenge* OR facilitator* OR obstacle*):ab,ti,kw OR (evaluat* OR difficult* OR enable* OR promot*):ti) **AND** ('dementia'/de OR 'Alzheimer disease'/exp OR (dement* OR Alzheimer*):ab,ti,kw)

**Web of Science**

TS=(((underrepresent* OR disadvant*) NEAR/2 (group* OR people* OR person OR persons OR patient* OR elder* OR client* OR adult*)) OR minorit* OR refugee* OR immigrant* OR migrant* OR hard-to-reach* OR ethnic* OR racial* OR race* OR ((social*) NEAR/2 (disadvant*))) AND TS=( dement* OR Alzheimer*) AND (TS=(barrier* OR challenge* OR facilitator* OR obstacle*) OR TI=(evaluat* OR difficult* OR enable* OR promot*))

**CINAHL**

(MH "Minority Groups" OR MH "Ethnic Groups+" OR MH "Refugees+" OR MH "Transients and Migrants" OR TI(((underrepresent* OR disadvant*) N3 (group* OR people* OR person OR persons OR patient* OR elder* OR client* OR adult*)) OR minorit* OR refugee* OR immigrant* OR migrant* OR hard-to-reach* OR ethnic* OR racial* OR race* OR ((social*) N3 (disadvant*))) OR AB(((underrepresent* OR disadvant*) N3 (group* OR people* OR person OR persons OR patient* OR elder* OR client* OR adult*)) OR minorit* OR refugee* OR immigrant* OR migrant* OR hard-to-reach* OR ethnic* OR racial* OR race* OR ((social*) N3 (disadvant*)))) **AND** (TI(barrier* OR challenge* OR facilitator* OR obstacle* OR evaluat* OR difficult* OR enable* OR promot*) OR AB(barrier* OR challenge* OR facilitator* OR obstacle*)) **AND** (MH "Dementia" OR MH "Alzheimer's Disease" OR TI(dement* OR Alzheimer*) OR AB(dement* OR Alzheimer*))

**Global Index Medicus**

(MH:"Minority Groups" OR MH:"Ethnic and Racial Minorities" OR MH:"Minority Health" OR MH:"Refugees" OR MH:"Transients and Migrants" OR TW:"underrepresented" OR TW:"disadvantaged" OR TW:minorit* OR TW:refugee* OR TW:immigrant* OR TW:migrant* OR TW:"hard to reach" OR TW:ethnic* OR TW:racial* OR TW:race*) AND (MH:"Evaluation Study" OR MH:"Evaluation Studies as Topic"* OR TW:barrier* OR TW:challenge* OR TW:facilitator* OR TW:obstacle* OR TI:evaluat* OR TI:difficult* OR TI:enable* OR TI:promot*) AND (MH:"Dementia" OR MH:"Alzheimer Disease" OR TW:dement* OR TW:Alzheimer*)
